# Multiple biomarkers of sepsis identified by novel time-lapse proteomics of patient serum

**DOI:** 10.1371/journal.pone.0222403

**Published:** 2019-09-30

**Authors:** Nobuhiro Hayashi, Syunta Yamaguchi, Frans Rodenburg, Sing Ying Wong, Kei Ujimoto, Takahiro Miki, Toshiaki Iba

**Affiliations:** 1 School of Life Science and Technology, Tokyo Institute of Technology, Ookayama, Meguro-ku, Tokyo, Japan; 2 Graduate School of Bioscience and Biotechnology, Tokyo Institute of Technology, Ookayama, Meguro-ku, Tokyo, Japan; 3 Nihon University Surugadai Hospital, Kanda-Surugadai, Chiyoda-ku, Tokyo, Japan; 4 Department of Emergency and Disaster Medicine, Juntendo University, Hongo, Bunkyo-ku, Tokyo, Japan; Istituto di Ricovero e Cura a Carattere Scientifico Centro di Riferimento Oncologico della Basilicata, ITALY

## Abstract

Serum components of sepsis patients vary with the severity of infection, the resulting inflammatory response, per individual, and even over time. Tracking these changes is crucial in properly treating sepsis. Hence, several blood-derived biomarkers have been studied for their potential in assessing sepsis severity. However, the classical approach of selecting individual biomarkers is problematic in terms of accuracy and efficiency. We therefore present a novel approach for detecting biomarkers using longitudinal proteomics data. This does not require a predetermined set of proteins and can therefore reveal previously unknown related proteins. Our approach involves examining changes over time of both protein abundance and post-translational modifications in serum, using two-dimensional gel electrophoresis (2D-PAGE). 2D-PAGE was conducted using serum from *n* = 20 patients, collected at five time points, starting from the onset of sepsis. Changes in protein spots were examined using 49 spots for which the signal intensity changed by at least two-fold over time. These were then screened for significant spikes or dips in intensity that occurred exclusively in patients with adverse outcome. Individual level variation was handled by a mixed effects model. Finally, for each time transition, partial correlations between spots were estimated through a Gaussian graphical model (GGM) based on the ridge penalty. Identifications of spots of interest by tandem mass spectrometry revealed that many were either known biomarkers for inflammation (complement components), or had previously been suggested as biomarkers for kidney failure (haptoglobin) or liver failure (ceruloplasmin). The latter two are common complications in severe sepsis. In the GGM, many of the tightly connected spots shared known biological functions or even belonged to the same protein; including hemoglobin chains and acute phase proteins. Altogether, these results suggest that our screening method can successfully identify biomarkers for disease states and cluster biologically related proteins using longitudinal proteomics data derived from 2D-PAGE.

## Introduction

The severity of sepsis varies significantly with the inflammatory response and the extent of organ dysfunction. Severe cases of sepsis during which hypotension continues even after adequate fluid resuscitation, is classified as septic shock [1]. Mortality rates of sepsis are as high as 20–30% and early initiation of proper treatment may greatly reduce the mortality rate [2–4].

Current diagnosis of sepsis and the treatment decisions are primarily based on the Sequential Organ Failure Assessment (SOFA) and Quick SOFA (qSOFA) [5, 6], but these are known to lack sensitivity and accuracy. C-reactive protein (CRP), interleukin-6 (IL-6), procalcitonin (PCT) and other biomarkers are also used to detect sepsis [7, 8], but none of these can adequately predict the outcome of sepsis [9, 10]. CRP is most commonly used as an indicator of infection, but it is also elevated in certain other conditions. While IL-6 is considered to be an important inflammatory cytokine, its usefulness in diagnosing sepsis is still controversial [11, 12]. PCT is a more recently proposed clinical target, albeit still controversial due to its elevation during non-infectious inflammation, e.g. following surgery or trauma [13]. Thus, while the call for novel biomarkers has led to several new suggestions, the accuracy is still considered by many to be insufficient.

The simplified acute physiology score II (SAPS II), attempts to address this by relying on a multitude of biomarkers. This yields far better performance than a single biomarker, but the score also takes considerable time to determine. Others have suggested that more effective treatment can be provided by dividing patients into groups [14–16], but this splitting comes at the cost of statistical power.

Systems biology attempts to model the cell as a whole and may therefore prove to be a superior method for distinguishing between disease states. The most popular branch of systems biology is genomics: the large scale study of genes. Cells are systems composed of proteins, and their conditions are essentially dependent on the relative amounts and states of these proteins (e.g. post-translational modifications). While genomics is among the most powerful approaches to profiling cells, the amount of a particular protein in a cell is affected by the efficiency of translation, post-translational processing, and degradation rates in the cell. Therefore, the actual amounts of proteins are poorly reflected by gene expression alone. Furthermore, current protein states, such as post-translational modifications (e.g. phosphorylation), cannot be investigated by genomics.

Contrastingly, proteomics directly analyzes proteins, enabling determination of both the amounts and states of the protein components of cells. Among proteomics techniques, two-dimensional gel electrophoresis (2D-PAGE) is the most powerful method for examining amounts and states of proteins directly. However, data quality is highly dependent on operation. In addition, as the procedure requires considerable skill and time, it is difficult to obtain large amounts of high-quality data. This practical limitation has prevented the widespread use of 2D-PAGE for clinical diagnosis as of yet, in spite of its promising potential for profiling cells.

Recently, we have developed a high-performance, two-dimensional polyacrylamide gel electrophoresis (HP-2D-PAGE) technique characterized by high throughput, high sensitivity, and high reproducibility [17]. In this procedure, higher stability of the electric field, voltage and temperature of both the first and second dimensional separation was achieved by drastically reducing the size of the gels ordinarily used for 2D-PAGE. This enhanced stability directly relates to the extent to which spots on the gel are in focus. Loss of focus results in deterioration of resolution of the spots and sensitivities of the signals. Additional improvements were achieved by ensuring the size and thickness of the filter papers on electrodes for the first dimension of separation were large and thick enough to absorb all salts accumulating at the electrodes. A final improvement was observed by optimizing the amount of total protein on the gels, depending on the type of sample used. As for human serum, this amount was optimized by maximizing discernable spots on the gels. Although this does not necessarily increase the total number of the spots compared with the conventional methods, the performance in terms of high throughput capacity, high sensitivity, and high reproducibility was improved sufficiently to enable practical applications.

For clinical applications, high dimensional serum data can be obtained in a short period from small amounts of specimen. The quality of the data must be high enough to enable comparisons between subjects. Our technique can rapidly provide proteomics data that meets the criteria necessary for clinical use. Nevertheless, realization of clinical proteomics using HP-2D-PAGE is still limited by the challenge of how to make the best use of the resulting complex 2D-PAGE image data.

In this study, changes in spot intensity over time were investigated, using 2D-PAGE images obtained from serum samples collected from sepsis patients. The interactions between these spots were then investigated using a network of partial correlations. It is likely that spots connected tightly in such a network function collectively in carrying out biological responses. This method enables identification of both groups of proteins responsible for known biological functions, and groups responsible for lesser known functions. In case of the latter, the function may be postulated based on their association with the disease in question. Furthermore, if spots of proteins with unknown functions are found to belong to groups with known biological functions, then this could enable elucidation of previously unknown protein functions. This is the first study to investigate the applicability of clinical proteomics diagnosis using high-quality 2D-PAGE image data. Moreover, there have been no reports of proteomic diagnostic techniques using samples collected over time yet.

## Experimental procedures

### Experimental design

Serum samples were collected from *n = 20* sepsis patients recruited from 1^st^ of May 2012 to 30^th^ September 2014, whose Sequential (Sepsis-Related) Organ Failure Assessment (SOFA) scores were raised more than 2.0 points at the Nihon University Hospital in accordance with the regulations of the ethics committee [5]. Clinical and pathological data of the patients included in this study are also shown in Table 1. Written informed consent was obtained from each patient before inclusion in the study. The patient group included both males and females, and the distribution of age was 35 to 79 years as shown in Table 1. 60 μL of serum was used per observation in the experiments. This study has been approved by the ethical committee of Nihon University, Juntendo University and Tokyo Institute of Technology under the approval numbers 120403, 2013087, and 2016069.

**Table 1:** Clinical and pathological data of the patients.

Patient ID	Age	Sex	Outcome of 14 days	Outcome of 28 days
**No.1**	52	F	L	L
**No.2**	74	F	D	D
**No.3**	79	M	D	D
**No.5**	45	F	L	L
**No.8**	53	M	L	L
**No.9**	72	M	L	L
**No.17**	51	F	L	L
**No.18**	47	M	L	L
**No.19**	78	F	L	L
**No.20**	64	M	L	L
**No.21**	42	F	L	L
**No.22**	75	M	L	D
**No.23**	69	F	D	D
**No.24**	64	F	L	L
**No.25**	35	M	L	D
**No.27**	46	M	L	L
**No.28**	71	M	L	L
**No.29**	65	M	L	L
**No.30**	36	M	L	L
**No.31**	59	M	L	L

Information such as patient ID, age, sex of patients and treatment outcome after 14 and 28 days are included. “F” represents female; “M” represents male. “L” indicates survival up to the specified time; “D” indicates non-surviving.

### Depletion of high-abundance proteins in serum

An Aurum Serum Protein Mini Kit (Bio-Rad) was used to deplete albumin and IgG, which is otherwise present in large amounts in serum, cluttering the gels. This procedure was performed twice in order to increase the efficiency of abundant protein depletion. Later, to further enhance the visibility of lower abundance proteins, the samples were treated with a Seppro IgY14 protein kit (Sigma-Aldrich), depleting 14 other highly abundant proteins, including: albumin, IgG, alpha 1-antitrypsin, IgA, IgM, transferrin, haptoglobin, a2-macroglobulin, fibrinogen, complement C3, a1-acid glycoprotein, high density lipoprotein (alipoproteins A-I and A-II) and low density lipoproteins (mainly alipoprotein B).

### 2D-PAGE

For accurate quantification, the amount of protein in each sample was determined using a 2-D Quant Kit (GE Healthcare). Removal of impurities and desalination of samples was carried out using a 2-D Clean-Up Kit (GE Healthcare).

Treated samples of 50 μg were dissolved in Destreak rehydration solution (GE Healthcare) and complete uptake of sample by Immobiline DryStrips (7 cm, pH 3–10) was allowed for at least 18 h at room temperature. Isoelectric focusing (IEF) as the first dimension was carried out using the rehydrated strips on a Multiphor II system (GE Healthcare) linked to a cooling circulator (Julabo). Filter papers on the electrodes with twice the regular size and thickness were used. IEF was performed by gradually increasing the voltage under these conditions: (i) 0–300 V, 1 min; (ii) 300 V, 1.5 h; (iii) 300–3500 V, 1.5 h and (iv) 3500 V, 500 hours (= ∞).

Second dimension separation was carried out on a discontinuous SDS gel system as first described by Laemmli [18] using a NuPAGE 4–12% Bis-Tris Z00m Gel (Thermo Fisher Scientific). The resulting gels were stained with the fluorescent dye: SYPRO Ruby Protein Gel Stain (Thermo Fisher Scientific).

Fluorescent images were acquired from the stained gels using a Typhoon FLA 9500 apparatus with Ettan DIGE imager software (GE Healthcare), and the images were analyzed using Image Master 2D Platinum 7.0 software (GE Healthcare).

### In-gel digestion

Excised protein spots were in-gel digested with trypsin according to a modified procedure [19]. Summarized, spots on gels that had undergone 2D-PAGE were excised and incubated in solution A (200 mM ammonium bicarbonate, NH4HCO3 [pH 8.1], 50% acetonitrile, ACN) for 20 min at room temperature. Next, the excised spots were incubated in solution B (100 mM NH4HCO3 [pH 8.1], 100 mM DTT) for 30 min at 60°C, after which they were left in ACN for 5 min at room temperature followed by trypsin solution (20 μg/mL Trypsin Gold [Promega], 0.2 mM HCl, 40 mM NH4HCO3 [pH 8.1], 5 mM CaCl2, 10% ACN) for 16 h at 37°C. Trypsin degradation products from gels processed in this manner were extracted in stages using ultrapure water (Wako Pure Chemical Industries), 0.1% trifluoroacetic acid TFA-60% ACN, 0.1% TFA-80% ACN, and 0.1% TFA-100% ACN.

### Mass spectrometry analysis and protein identification

Proteins belonging to spots of interest on the gels were identified by comparing the amino acid sequences of trypsin digestion products, obtained in analysis using a liquid chromatography–tandem mass spectrometry (LC/MS/MS) system with an existing database.

LC-MS/MS analyses were performed using a system that consists of an inlet system (Acquity UPLC, Waters) and a Quadrupole-time-of-flight (Q-TOF) mass spectrometer (SYNAPT G2 HDMS, Waters) as follows: The in-gel digested samples were injected to the inlet system with C18 column (75-μm diameter; 150-mm length; particle size, 1.7 μm) at a flow rate of 0.5 μl/min. Solutions used for the LC were ultrapure water containing 0.1% formic acid (A) and ACN containing 0.1% formic acid (B). A gradient program of 1% B (0 min), 40% B (30 min), and 95% B (31 min) was used. Samples separated by LC were introduced into the electrosprayer for MS/MS analyses in order. The mass spectrometer was operated in positive ionization mode, and a capillary voltage of 3.0–3.2 kV and a cone voltage of 30 V were utilized. The inlet LC flow was nebulized using nitrogen gas (700 L/h). Argon gas was used for collision-induced dissociation. The system was equipped with an integral LockSpray unit with its own reference sprayer that was controlled automatically by the acquisition software to collect a reference scan every 10 s. The reference for LockSpray was Glu-fibrinopeptide B (Sigma) at a concentration of 200 fmol/μL. The reference solution was introduced into the lock mass sprayer at a flow rate of 0.5 μL/min. A single lock mass calibration at m/z 785.8426 in positive ion mode was used during analysis. MS data were acquired over the mass range of 50–1990 Da with a scan time of 1 s and a detector voltage of 1800 V. The MS data obtained were searched against Swiss-Prot database human (*Homo sapiens*) through the application of the search program ProteinLynx Global Server (PLGS) (Waters), and reliability of the results were judged using the PLGS scores [20–22].

### Statistical analysis

After segregating spots using the watershed transform in ImageMaster 2D Platinum, spot intensities were analyzed in R [23]. Intensities of spots absent from gels were imputed with the background noise. For this exploratory study, only the 2-week and 4-week survival of patients was available. Hence, the 4-week survival was used as a proxy for good or poor outcome. In addition, we assessed which of the identified proteins showed significant (non-linear) change over time, so that these might be the subject of future studies with greater sample sizes and more detailed records of short-term or long-term outcome.

A linear mixed effects model was used to assess significant changes in log-intensity over time, using the package lme4 [24]. An interaction between time and 4-week survival was estimated and time was encoded as a factor to allow the model to pick up non-linear changes over time. Spot intensity is non-negative, with variance growing with the mean (higher intensity spots display considerably larger variance), so a logarithmic transformation was used. This yielded approximate residual normality and allowed for similar comparison of both small and large spots. A random intercept for patients was estimated to address the temporal dependence between patients’ measurements. A simpler model using a mixed effects smoothing spline was also considered, but at the time of writing this paper, no implementation allowing for interactions between fixed effects was readily available. Comparisons were restricted to adjacent time points and confidence intervals were obtained, using the profile-likelihood method.

Proteins potentially involved in sepsis progression were considered as such based on a search for dips and spikes in intensity exclusively observed in patients with poor outcome. That is to say, a significant interaction term between two adjacent time points and the 4-week survival, directly followed by a significant change in the opposite direction.

Next, protein-protein interactions were assessed using a separate model for significant (non-linear) changes over time. Marginal significance at ɑ = 0.05 was used to select proteins for which there was at least one significant change between adjacent time points. These proteins were then used to calculate the difference between time points and estimate an inverse covariance matrix of these differences, using the ridge regularized implementation in the package rags2ridges [25–26]. Exact leave-one-out cross validation (LOOCV) was used to select regularization scalar λ. A fused penalty for precision matrices estimated from different time points was also considered, but the fusion parameter was optimal at near-zero values and therefore omitted. A local false discovery rate of 0.05 was used to select non-zero elements of the standardized inverse covariance matrix, as proposed by Schäfer & Strimmer 2005 [27]. This yielded a sparse matrix of partial correlations, which was used to construct a heatmap of partial correlations and a conditional independence network to assess relationships between proteins observed in sepsis progression over time.

### Western blotting

In order to validate some of the protein biomarkers identified in the current study, Western blotting was performed. Using a reference sample from the surviving (L) and adverse outcome group (D), separation of proteins from 10 μg of serum sample was performed by 8–12% sodium dodecyl sulfate polyacrylamide gel (SDS-PAGE). Precision Plus Protein WesternC Standard (Bio-Rad) was used as protein standard. Proteins were then allowed to transfer to a polyvinylidene difluoride (PVDF) membrane at 200 V, 0.12 A for an hour at room temperature. The blot was then probed for the proteins of interest. Primary antibodies used include anti-haptoglobin antibody (ab95846, Abcam) and anti-complement factor B antibody (ab133765, Abcam) to probe haptoglobin protein and complement factor B protein in the sample, respectively. Blocking buffer consisting of 5% skim milk/TBS-T (0.1% Tween 20) was used for dilution of the antibodies according to the manufacturer’s recommendation. Primary antibody staining was performed overnight at 4°C. Secondary antibody staining was then performed using HRP Goat Anti-Rabbit Goat IgG (AB_2795955, Southern Biotech) for an hour at room temperature. Detection of proteins was done using Merck Immobilon Western Chemiluminescent HRP substrate (Fisher Scientific).

## Results

### Identification of spots exhibiting substantial changes in signal intensity over time

**Fig 1:**
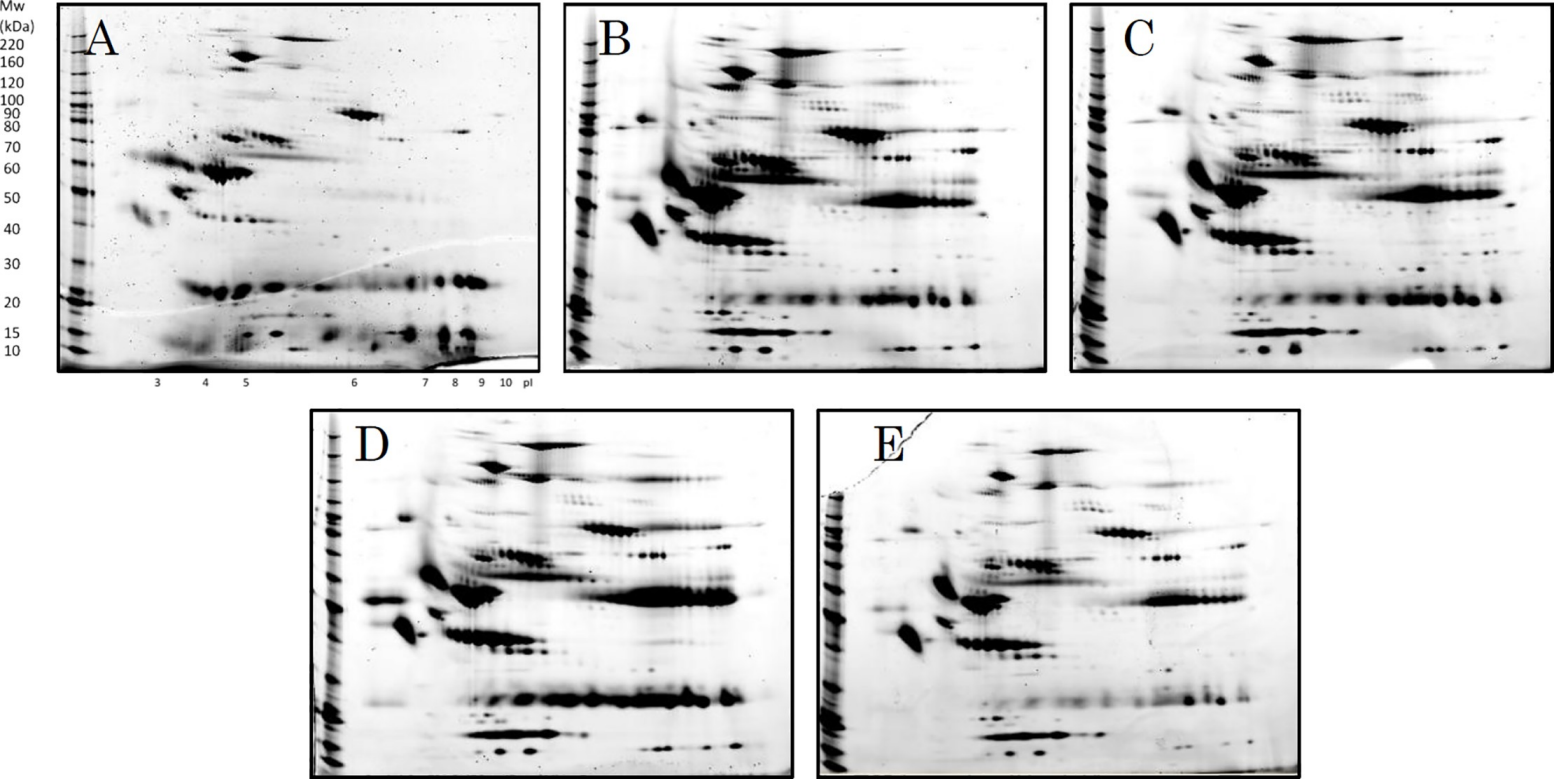
Two-dimensional electrophoresis images of the serum of sepsis patient No. 1. Serum as collected A) at the start of treatment, B) after 1 day, C) after 3 days, D) after 5 days, and E) after 7 days. The data for the remaining 19 patients are included in the supplementary files (S1–S19 Figs).

**Fig 2:**
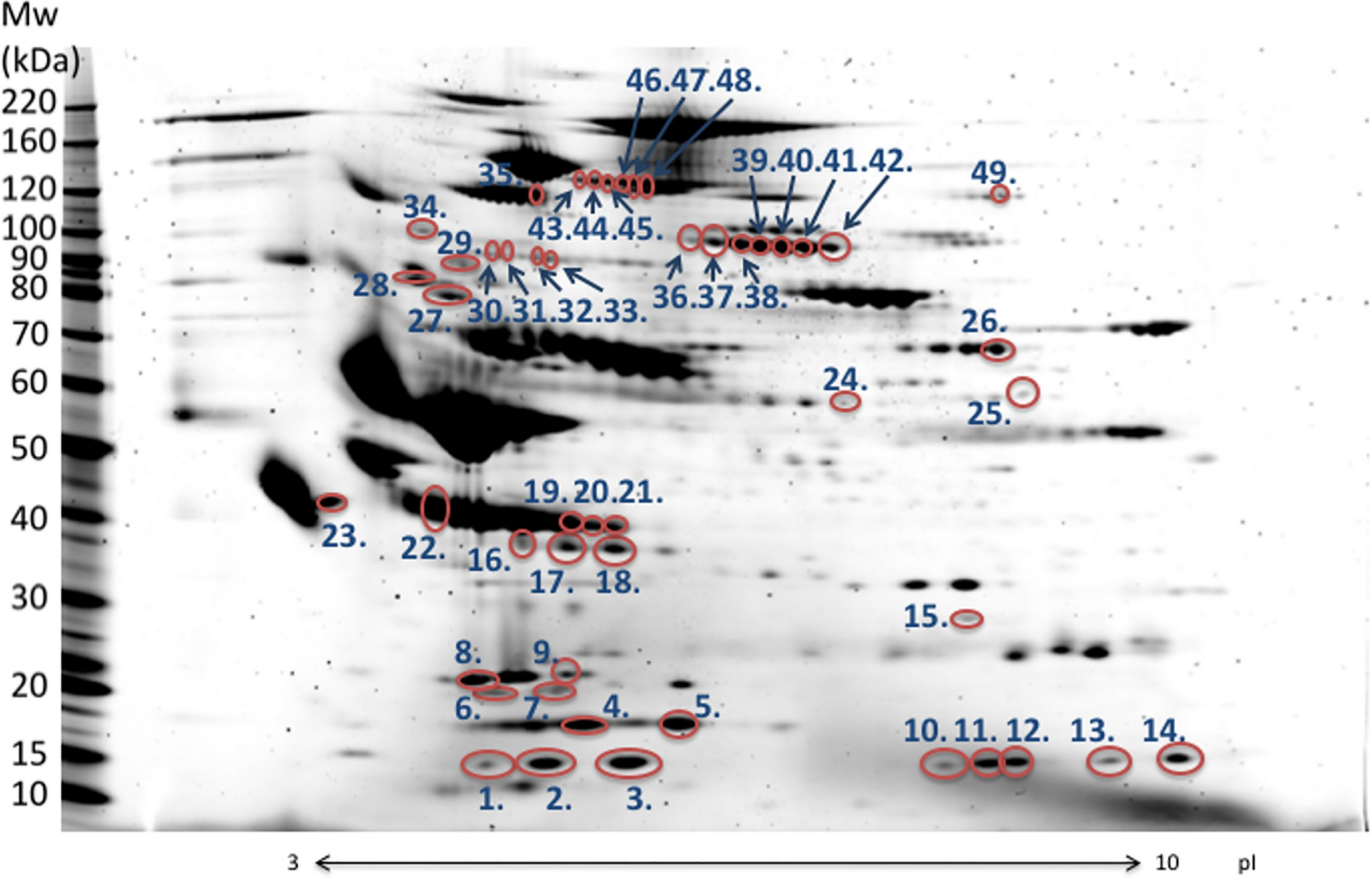
Two-dimensional electrophoresis image of patient serum. Serum from sample no. 9 (A, start of the treatment) was used. Molecular weight is indicated on the vertical axis, and isoelectric point is indicated on the horizontal axis. Among spots which signal changed substantially over time, those that were successfully identified are circled in red and numbered.

2D-PAGE was performed using five measurements over time, corresponding to the first, second, third, fifth, and seventh day after admittance (Fig 1). For each patient, by comparing 2D-PAGE images with images from the day before, spots with signals that had changed in intensity by at least two-fold (increase) or half-fold (decrease) were extracted. A total of 83 unique spots were obtained this way. Using tandem mass spectrometry, 49 out of these 83 spots were successfully identified with reliable PLGS scores (Fig 2, Table 2). The remaining spots are simply named 1 to 34 in the tables and figures.

**Table 2:** Identification of spots which signals changed substantially over time.

No.	Accession no.	Protein Description	Score
**1**	A6XGL1	Transthyretin OS Homo sapiens PE 2 SV 1	687
**2**	A6XGL1	Transthyretin OS Homo sapiens PE 2 SV 1	5933
**3**	A6XGL1	Transthyretin OS Homo sapiens PE 2 SV 1	25552
**4**	P02057	Hemoglobin beta 1 and beta 2 chains	393
**5**	D9YZU5	Hemoglobin beta OS Homo sapiens GN HBB PE 3 SV 1	7112
**6**	P68871	Hemoglobin subunit beta OS Homo sapiens GN HBB PE 1 SV 2	6679
**7**	P01944	Hemoglobin alpha chain	577
**8**	G3V1N2	HCG1745306 isoform CRA a OS Homo sapiens GN HBA2 PE 3 SV 1	833
**9**	J3QLC9	Haptoglobin Fragment OS Homo sapiens GN HP PE 3 SV 1	505
**10**	E7EVA3	Complement factor B OS Homo sapiens GN CFB PE 4 SV 1	104
**11**	P02741	C reactive protein OS Homo sapiens GN CRP PE 1 SV 1	1986
**12**	P00738	Haptoglobin OS Homo sapiens GN HP PE 1 SV 1	1998
**13**	Q6NSB4	HP protein OS Homo sapiens GN HP PE 2 SV 1	3925
**14**	Q0VAC5	HP protein OS Homo sapiens GN HP PE 2 SV 1	7042
**15**	P00738	Haptoglobin OS Homo sapiens GN HP PE 1 SV 1	5515
**16**	Q0VAC5	HP protein OS Homo sapiens GN HP PE 2 SV 1	7943
**17**	J3QR68	Haptoglobin Fragment OS Homo sapiens GN HP PE 3 SV 1	1755
**18**	H7C146	Microtubule associated serine threonine protein kinase 4 Fragment OS Homo sapiens GN MAST4 PE 4 SV	1393
**19**	B4DMA2	cDNA FLJ54023 highly similar to Heat shock protein HSP 90 beta OS Homo sapiens PE 2 SV 1	793
**20**	P00734	Prothrombin OS Homo sapiens GN F2 PE 1 SV 2	505
**21**	H7C5H1	Complement factor B Fragment OS Homo sapiens GN CFB PE 3 SV 1	224
**22**	P02741	C reactive protein OS Homo sapiens GN CRP PE 1 SV 1	1646
**23**	J3QR68	Haptoglobin Fragment OS Homo sapiens GN HP PE 3 SV 1	6176
**24**	E7EVA3	Complement factor B OS Homo sapiens GN CFB PE 4 SV 1	460
**25**	P01024	Complement C3 OS Homo sapiens GN C3 PE 1 SV 2	448
**26**	B4DNX0	cDNA FLJ51654 highly similar to von Willebrand factor OS Homo sapiens PE 2 SV 1	451
**27**	A7E2V2	Complement component 4A Rodgers blood group OS Homo sapiens GN C4A PE 2 SV 1	3049
**28**	A2BHY4	Complement component C4B Childo blood group OS Homo sapiens GN C4B 1 PE 4 SV 1	188
**29**	Q6U2F0	C4A2 Fragment OS Homo sapiens GN C4A PE 4 SV 1	285
**30**	F5GXS0	Complement C4 B OS Homo sapiens GN C4B PE 4 SV 1	3768
**31**	F5GXS0	Complement C4 B OS Homo sapiens GN C4B PE 4 SV 1	3509
**32**	B7Z5Q2	cDNA FLJ58075 highly similar to Ceruloplasmin EC 1 16 3 1 OS Homo sapiens PE 2 SV 1	2048
**33**	P02741	C reactive protein OS Homo sapiens GN CRP PE 1 SV 1	2806
**34**	B4DN17	cDNA FLJ61139 OS Homo sapiens PE 2 SV 1	69
**35**	P01023	Alpha 2 macroglobulin OS Homo sapiens GN A2M PE 1 SV 3	1811
**36**	P01023	Alpha 2 macroglobulin OS Homo sapiens GN A2M PE 1 SV 3	680
**37**	B0QYT1	Novel protein Fragment OS Homo sapiens GN RP4 756G23 6 002 PE 4 SV 1	48
**38**	P01023	Alpha 2 macroglobulin OS Homo sapiens GN A2M PE 1 SV 3	476
**39**	J3QLC9	Haptoglobin Fragment OS Homo sapiens GN HP PE 3 SV 1	406
**40**	P01031	Complement C5 OS Homo sapiens GN C5 PE 1 SV 4	45
**41**	P02741	C reactive protein OS Homo sapiens GN CRP PE 1 SV 1	425
**42**	E5RHP7	Carbonic anhydrase 1 Fragment OS Homo sapiens GN CA1 PE 4 SV 1	922
**43**	P01023	Alpha 2 macroglobulin OS Homo sapiens GN A2M PE 1 SV 3	185
**44**	B4E1Z4	Complement factor B OS Homo sapiens GN CFB PE 2 SV 1	129
**45**	B4E1Z4	Complement factor B OS Homo sapiens GN CFB PE 2 SV 1	2033
**46**	P00751	Complement factor B OS Homo sapiens GN CFB PE 1 SV 2	3152
**47**	E7EVA3	Complement factor B OS Homo sapiens GN CFB PE 4 SV 1	786
**48**	P01023	Alpha 2 macroglobulin OS Homo sapiens GN A2M PE 1 SV 3	3986
**49**	Q30KR1	Beta defensin 109 OS Homo sapiens GN DEFB109P1 PE 3 SV 1	595

“No.” represents spot number from Fig 2. “Accession no.” are obtained from UniProt database. “Score” indicates scores of ProteinLynx Global Server (PLGS).

### Identification of significant changes over time

**Fig 3:**
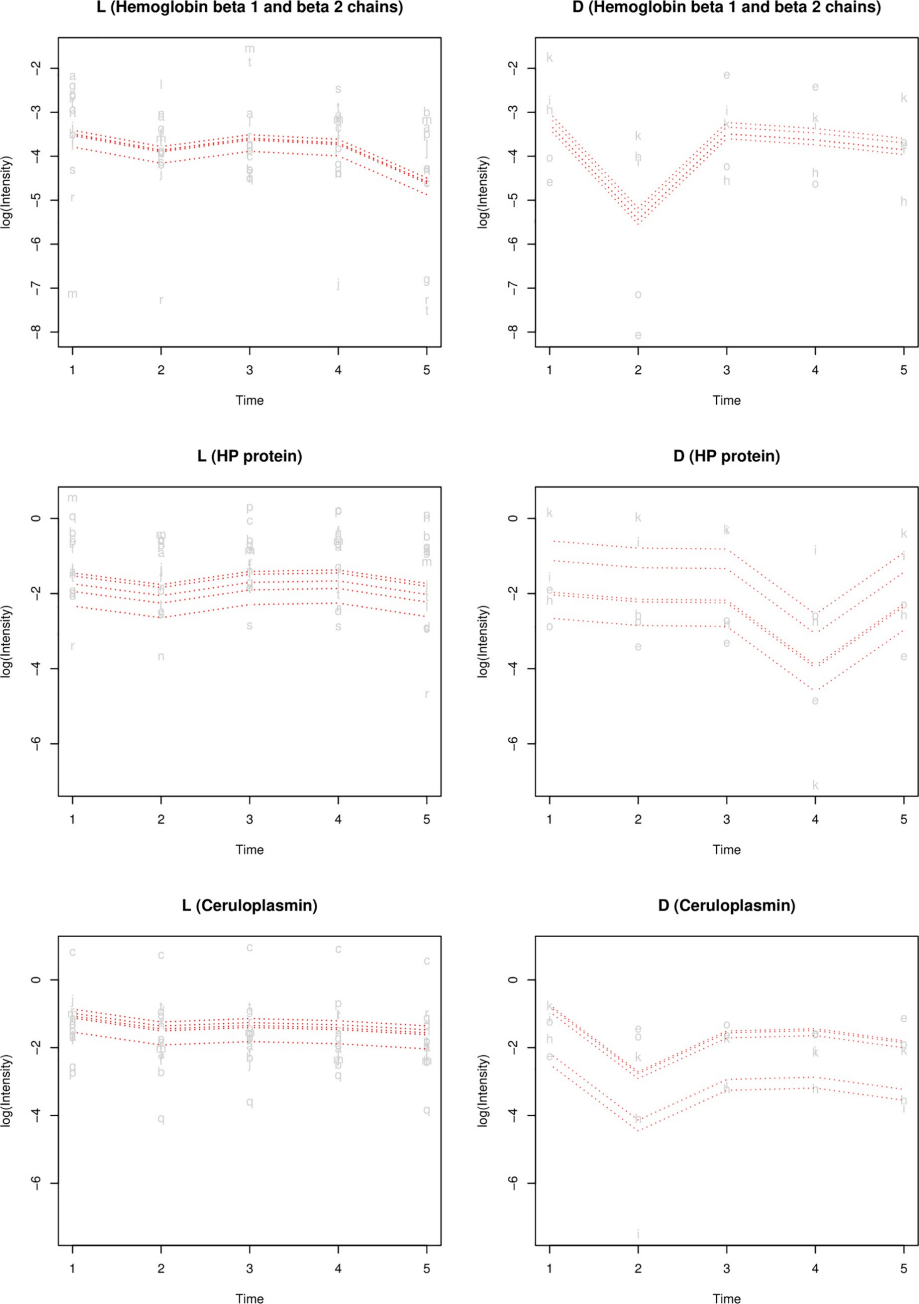
Significant changes in log-intensity of proteins over time from the spike-and-dip search. This includes the changes in protein expression of hemoglobin beta1 and beta 2 chains, haptoglobin and ceruloplasmin observed in the surviving group (L) and adverse outcome group (D).

Using the spike-and-dip search explained in the methods section, three proteins (hemoglobin beta 1 and beta 2 chains, haptoglobin and ceruloplasmin) were found to display significant dips exclusively in the poor outcome group (D) (Fig 3). In the surviving group (L), these proteins remained at relatively stable levels and showed no significant differences between adjacent time points.

### Protein-protein interactions

Protein-protein interactions were assessed by estimating a graph of partial correlations from the differences in log-intensities between time points, as described in the methods section. Partial correlations can be interpreted as the correlation between two variables after accounting for their correlation with all other variables. Whereas marginal correlations are affected by confounders, partial correlations have the advantage of showing only direct relationships, provided the confounders are also included in the calculation. Among others, partial correlations have previously been successfully used to create gene-gene interaction networks [28].

**Fig 4:**
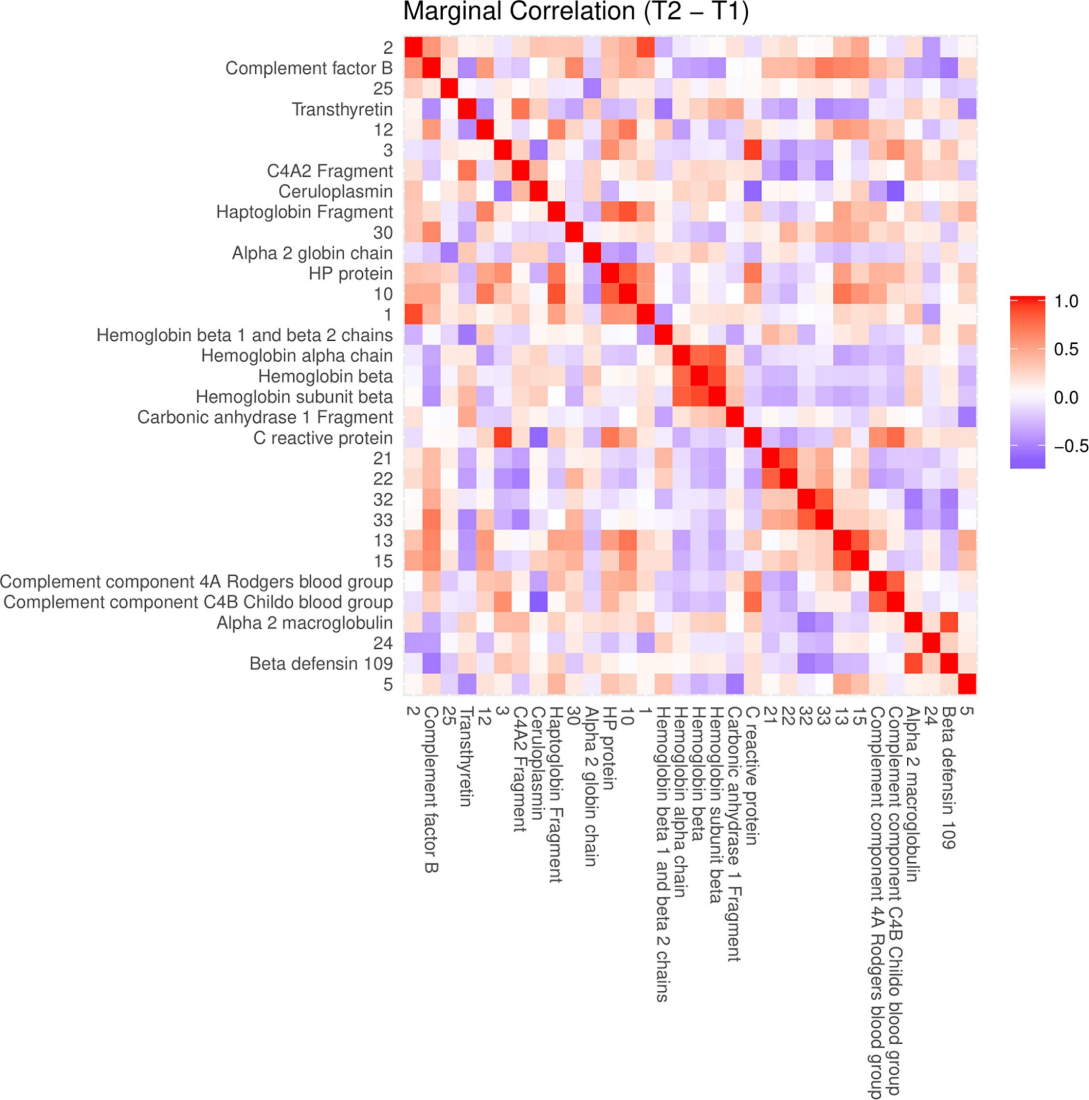
An ordinary heatmap of marginal correlations between differences in spot intensities from *t*_1_ to *t*_2_. Row and column order were set by hierarchical clustering using *f*(*x*) = 1−*cor*(*x*) as distance function. Color represents positive (red) or negative (blue) correlations. Heatmaps corresponding to the other time point transitions are included as supplementary figures (S20, S21 and S22 Figs).

**Fig 5:**
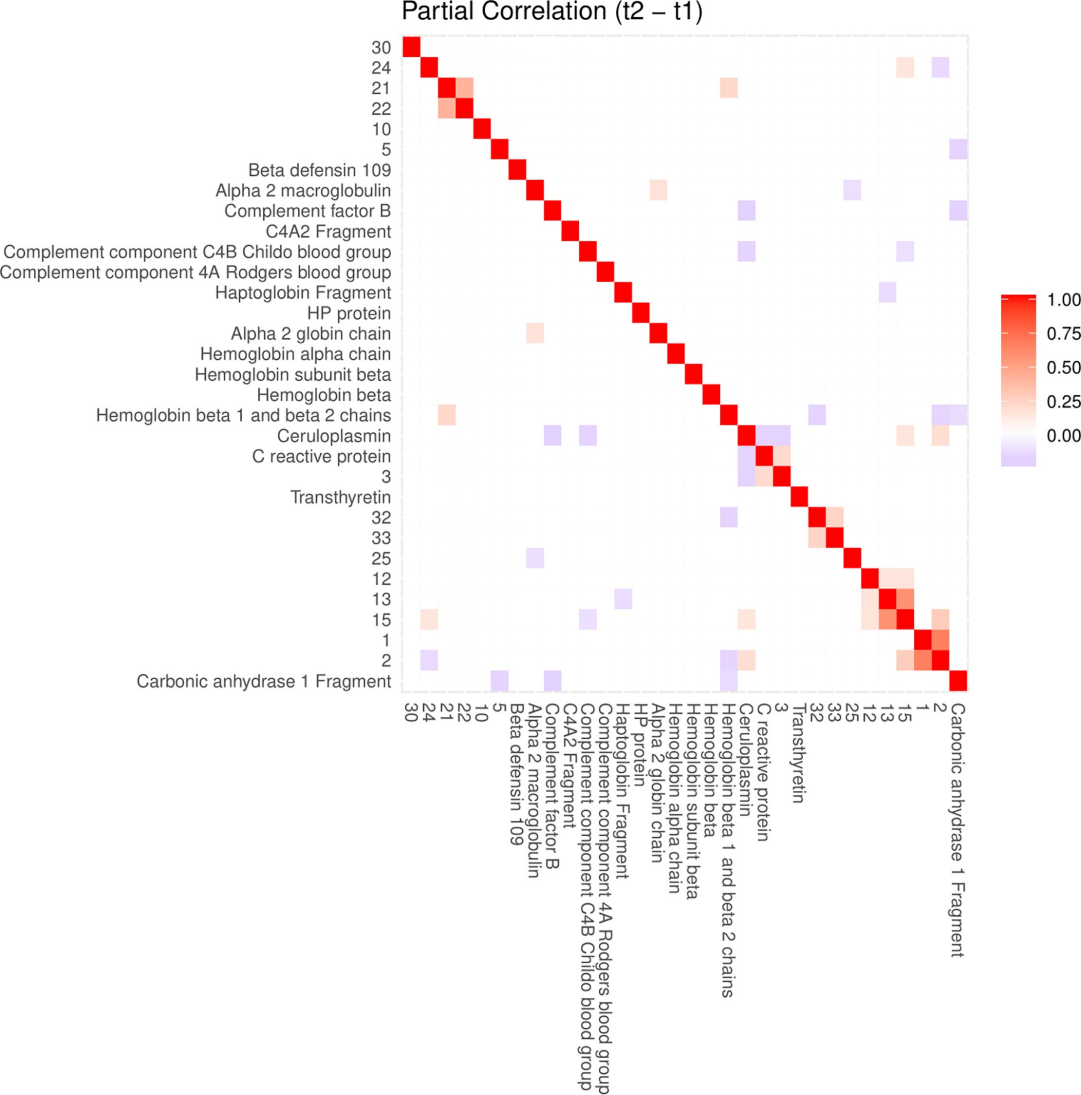
A heatmap of partial correlations between differences in spot intensities from *t*_1_ to *t*_2_. Contrary to the heatmap in Fig 3, a heatmap of partial correlations as depicted here is relatively uncluttered. Sparsity was achieved by estimating the support of the inverse covariance matrix through a local false discovery rate of 0.05 as described in the methods section. Row and column order were set by hierarchical clustering using *f*(*x*) = 1−*pcor*(*x*) as distance function. Heatmaps of partial correlations corresponding to the other time point transitions are included as supplementary figures (S23, S24 and S25 Figs).

**Fig 6:**
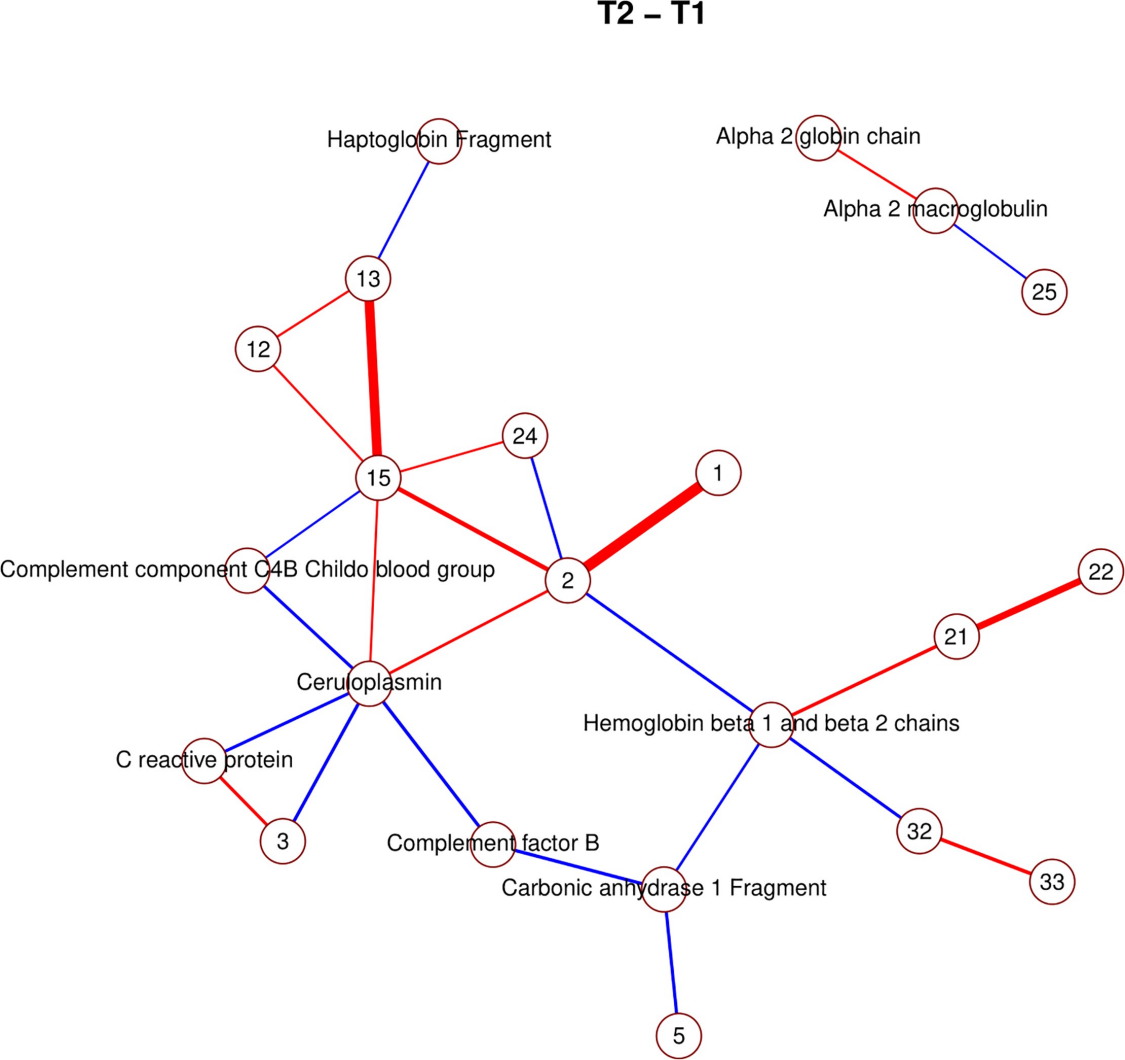
Non-zero partial correlations between differences in spot intensities from *t*_1_ to *t*_2_ displayed as a conditional independence network. Line width is proportional to the strength of the partial correlation and color represents positive (red) or negative (blue) partial correlation.

The pre-selection based on a mixed model yielded 39 proteins that changed significantly at least once between time points. The majority of significant changes (26) were observed between time points *t*_1_ and *t*_2_. Figs 4, 5 and 6 show, for time point transition *t*_1_→*t*_2_, the marginal correlations, partial correlations and a conditional independence network of partial correlations, respectively. By comparing the partial correlations with their more commonly used marginal counterparts, it is clear that the partial correlations provide a much less cluttered image than marginal correlations, by excluding all indirect relationships through lFDR thresholding. Heatmaps of the marginal and partial correlations of other time point transitions are included as supplementary figures.

**Fig 7:**
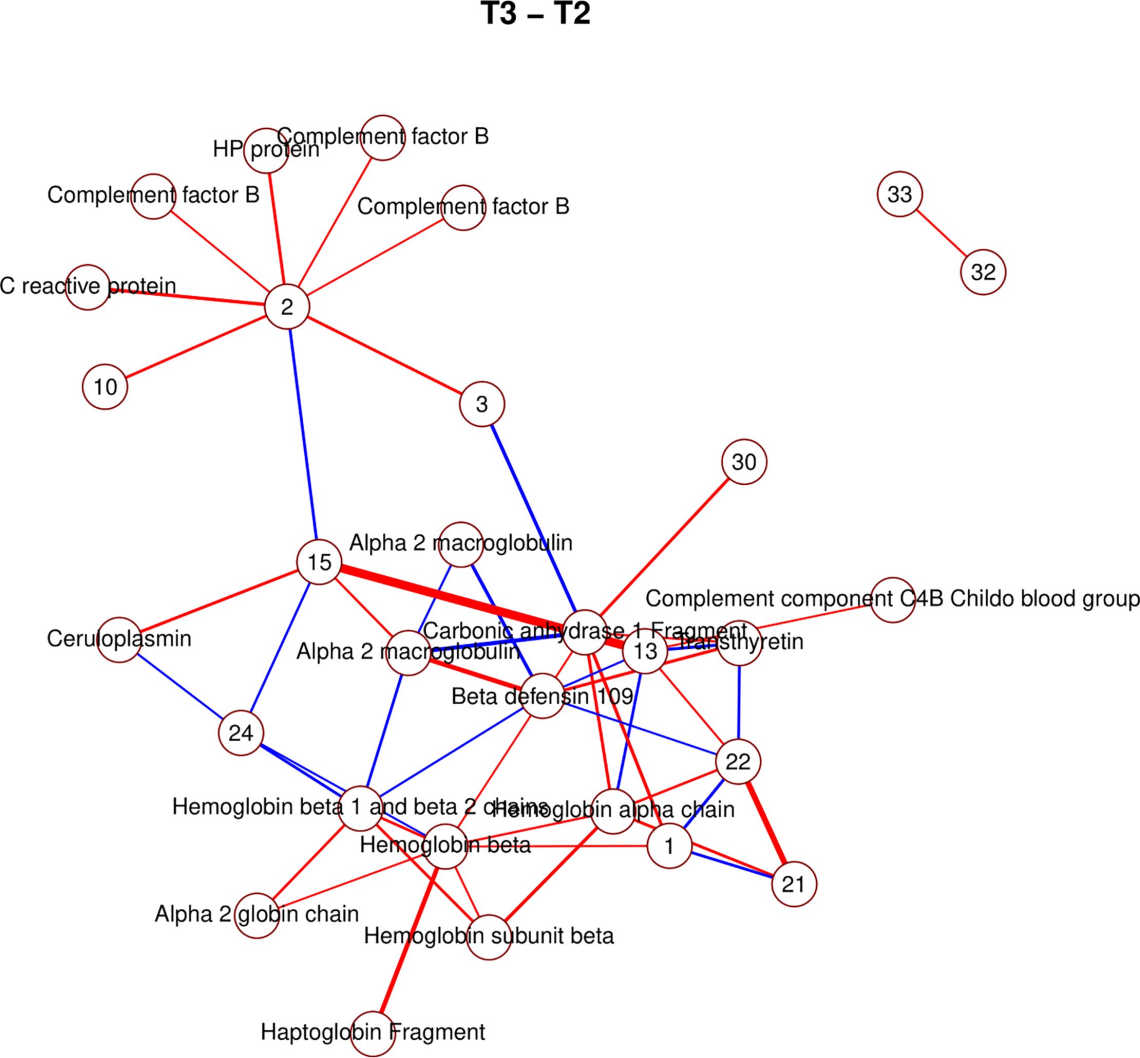
Non-zero partial correlations between differences in spot intensities from *t*_2_ to *t*_3_ displayed as a conditional independence network. Line width is proportional to the strength of the partial correlation and color represents positive (red) or negative (blue) partial correlation.

**Fig 8:**
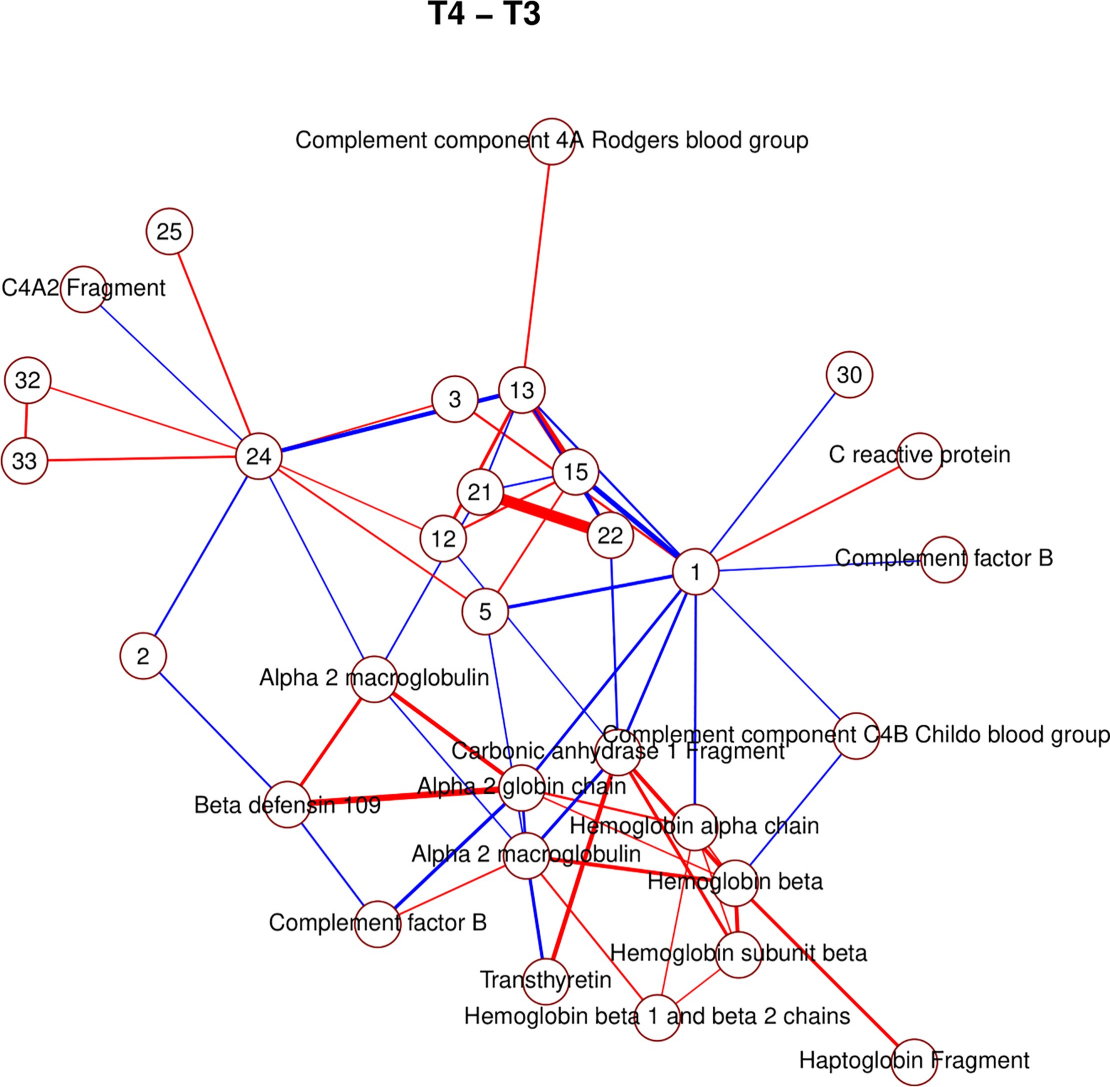
Non-zero partial correlations between differences in spot intensities from *t*_3_ to *t*_4_ displayed as a conditional independence network. Line width is proportional to the strength of the partial correlation and color represents positive (red) or negative (blue) partial correlation.

**Fig 9:**
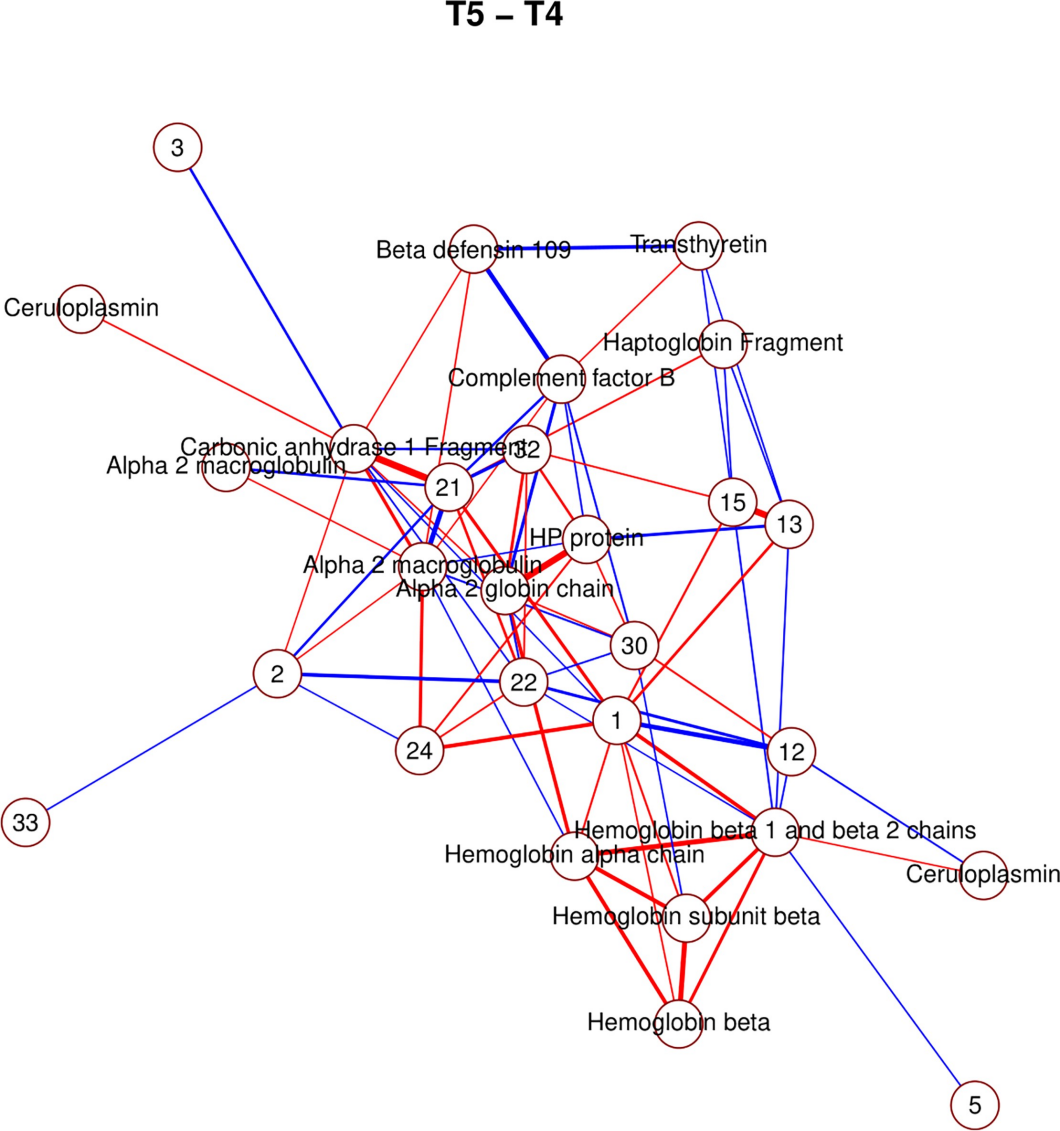
Non-zero partial correlations between differences in spot intensities from *t*_4_ to *t*_5_ displayed as a conditional independence network. Line width is proportional to the strength of the partial correlation and color represents positive (red) or negative (blue) partial correlation.

Due to the large amount of identified proteins, it is possible to observe several clusters of interacting spots corresponding to components of the same proteins (Figs 6, 7, 8 and 9; e.g. hemoglobin, alpha macroglobulin, complement components). This would seem to suggest that the network-based approach is successful in capturing meaningful biological interactions. In the mixed effects model, the majority of significant differences (26) were observed in the transition *t*_1_→*t*_2_, and this is also where the resulting graph is the clearest (Fig 6).

### Validation of the detected protein biomarkers

**Fig 10:**
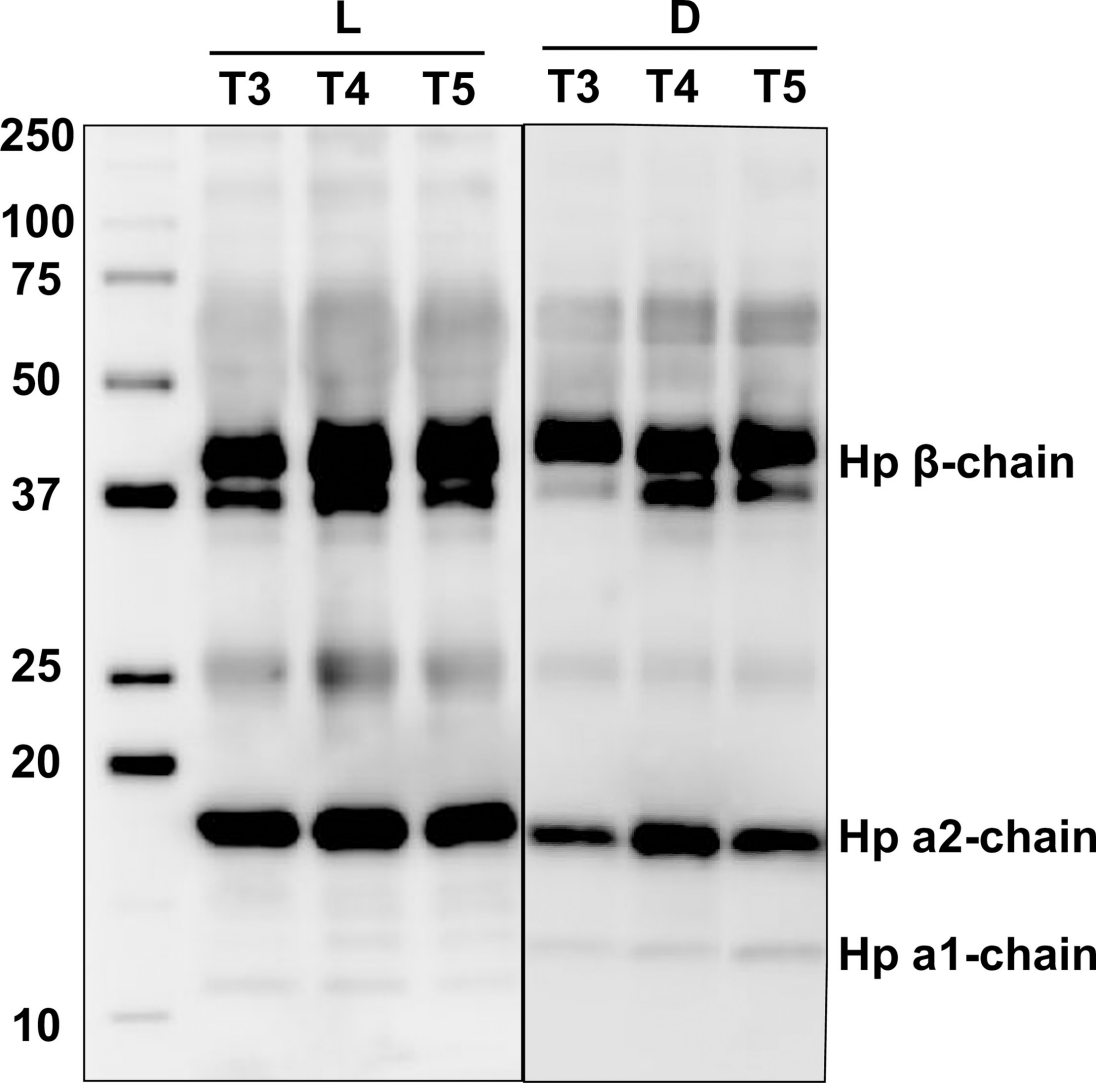
Detection of complement factor B protein in the surviving group (L) and adverse outcome group (D) by means of Western blotting. Two time points of each group were compared to confirm the overall change over time (*t*_1_→*t*_5_).

**Fig 11:**
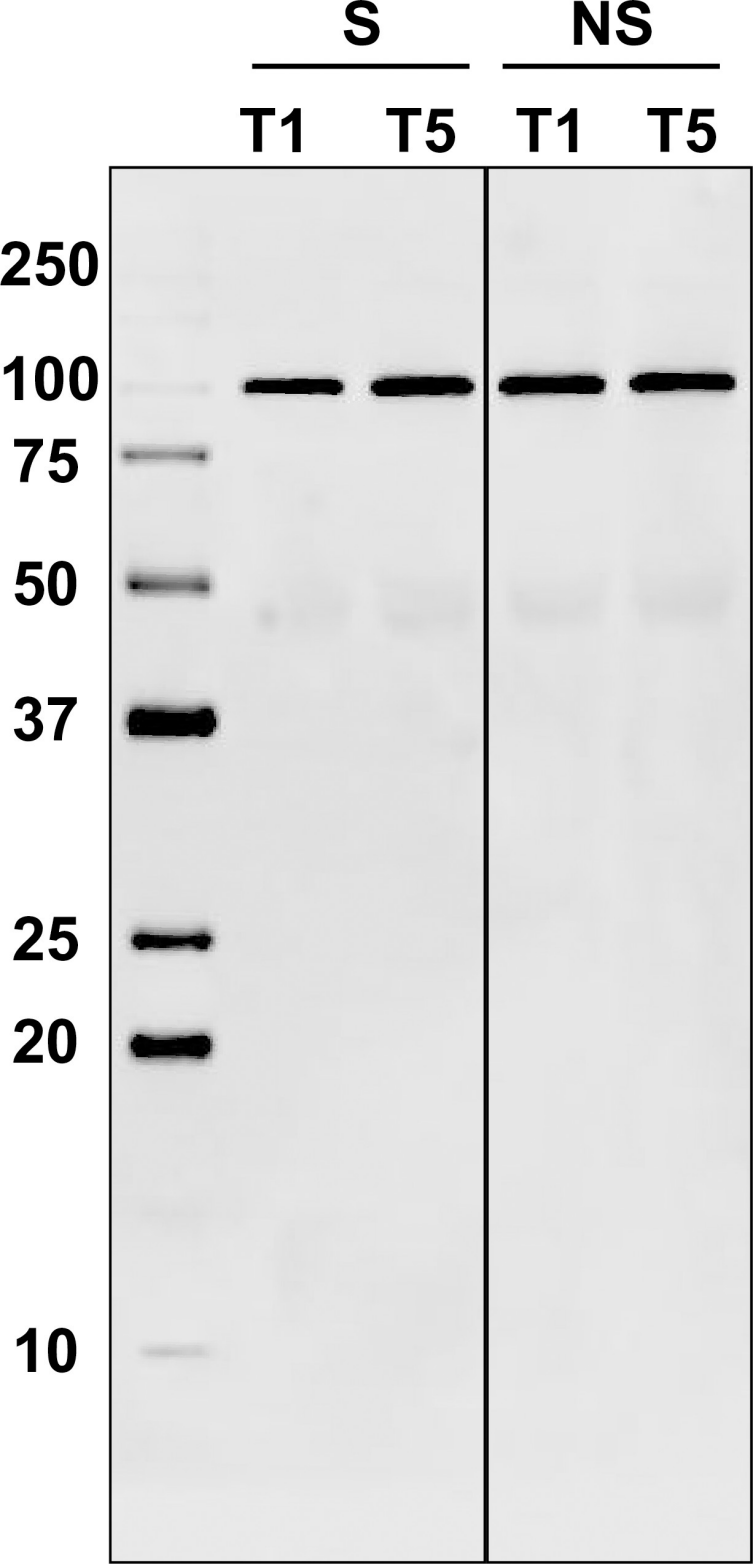
Detection of haptoglobin in the surviving group (L) and adverse outcome group (D) by means of Western blotting. Three time point of each group were compared to illustrate the progression over time (*t*_3_→*t*_4_→*t*_5_).

The identity of the protein biomarkers detected in the current study was subsequently confirmed by Western blotting (Figs 10 and 11). When comparing the levels of complement factor B protein between the surviving group (L) and adverse outcome group (D), the surviving group displayed an overall increase in intensity over time (from *t*_1_ to *t*_5_). Contrastingly, the intensity remained relatively unchanged in the poor outcome group (D).

Additionally, the detection of haptoglobin in both groups is shown in Fig 11, where Hp β-chain was detected at around 40 kDa, Hp α2-chain and α1-chain at 12–18 kDa. In the surviving group (L), an increase in haptoglobin levels from *t*_3_ to *t*_5_ can be observed, primarily in the Hp β-chain and Hp α2-chain. In the poor outcome group (D), however, Hp β-chain remained at an almost equal level from *t*_3_ to *t*_5_. While one could argue there appears to be an increase in Hp α2-chain from *t*_3_ to *t*_4_, this increase diminished again from *t*_4_ to *t*_5_.

## Discussion

This study employed a high-performance, two-dimensional polyacrylamide gel electrophoresis (HP-2D-PAGE) technique to study changes in the proteome during the progression of sepsis, a life-threatening condition which severity remains difficult to diagnose timely.

In 2D-PAGE, molecules that differ outside of phosphorylation or other post-translational modification have different electrical charges and so the same proteins can form spots in different positions. Hence, when the name of a given protein appears more than once, this strongly suggests that the abundance of that protein has not changed, but rather the state of its post-translational modification has changed. When 2D-PAGE images of such high resolution are attainable as illustrated in the current study, it is possible to identify various PTM sites as proteins of different isoforms and levels have been well-separated.

Several proteins identified in this study, including Transthyretin (TTR) and C-reactive protein (CRP), have already been used to diagnose sepsis clinically. CRP synthesis is promoted in the liver, and it is the protein most commonly used as an inflammatory indicator in sepsis and similar conditions [6]. TTR functions as a retinol-binding protein or a carrier of thyroxine, and in contrast to CRP, TTR is a negative acute phase protein which production is inhibited by inflammatory responses [29]. On the other hand, alpha 2-macroglobulin and the antibacterial peptide beta-defensin, are both also involved in immune responses. Neither of these have not been implicated in sepsis in previous studies.

Proteins like complement components play important roles in inflammation, including opsonization and lysis in the innate immune response. A trend towards increase in spot intensity of complement factor B was observed, mainly in the surviving group. This was then confirmed by means of western blotting (Fig 10), where an increase in complement factor B, exclusive to the surviving group (L) could be seen. This is thought to reflect the differences in immune system activation in different patients, where proper activation can have a detrimental effect on survival. These results suggest that the state of sepsis in terms of severity can be classified more precisely by focusing on the complement system, which closely reflects the state of the immune system as a whole.

Other proteins identified in this study, which are related to coagulation and thrombosis, include the von Willebrand factor (vWF) and prothrombin. In sepsis, acceleration of the blood coagulation and thrombus formation constitute one aspect of the host defense mechanisms [30, 31]. Prothrombin was detected in only some of the patients in the present study, but increased vWF was observed in many of the patients in the non-surviving group. vWF is produced constitutively as an unusually large multimer that is cleaved by a disintegrin and metalloproteinase with a thrombospondin type I motif, member 13 (ADAMTS13) and released into the blood. Interestingly, the vWF identified in this study was not mature-type vWF but the propolypeptide portion that is cleaved at maturity. vWF is a protein needed for hemostasis and for protecting and transporting coagulation factor VII [32]. It normally consists of 270 kDa subunits, but immediately after synthesis, it is composed of signal peptides, propolypeptides, and vWF subunits. When released into the blood, the signal peptides are removed, and dimers are formed with disulfide bonds at the C-terminus. The propolypeptides are then cleaved by furin, and dimers of subunits are formed via disulfide bonds at the N-termini to produce multiple polymers [33]. The spots identified in this study represented propolypeptides separated from mature vWF. These propolypeptides are released into the blood when inflammatory mediators activate endothelial cells, and they are known to positively correlate with the amounts of IL-6, IL-8, and IL-10 in sepsis patients [31–33]. However, unlike mature vWF, the physiologic function of the propolypeptide is essentially unknown. The surviving group exhibited decreased expression of vWF propolypeptide after 5 and 7 days, but no decrease was seen in the non-surviving group until the end of the study period. In the adverse outcome group, it was thought that the immune system was activated until the end, strongly suggesting that vWF propolypeptide plays a role in host defense in sepsis.

In the analysis of significant changes of proteins over time, our approach to biomarker detection yielded three proteins including hemoglobin beta 1 and 2 chains, haptoglobin as well as ceruloplasmin. These proteins displayed a significant and substantial dip in abundance between time points. Hemoglobin present in red blood cells has been shown to be free in serum along with hemolysis in severe sepsis. Free hemoglobin and free heme are both toxic, so elevated levels of haptoglobin, a scavenger of these substances, is a protective response of the body [34–36]. Since the systemic inflammation in sepsis causes a drop in blood pressure from widening blood vessels, it should come as no surprise that hemoglobin chains are associated with its progression. In this study, a sudden drop in hemoglobin beta 1 and 2 chains were observed in the poor outcome group. This can perhaps be explained by the drop in blood pressure from systemic inflammation in sepsis progression [37]. The significant dip observed exclusively in the adverse outcome group could prove to be valuable in assessing sepsis severity of patients.

Furthermore, a decrease in haptoglobin (HP) protein was also observed in this study. Since HP binds free hemoglobin that would otherwise accumulate following hemolysis, a sudden decline in HP protein (haptoglobin precursor) levels is indicative of hemolysis. Previous studies have also suggested the administration of haptoglobin as a therapeutic strategy against lethal sepsis and liver injury [38]. Major adverse kidney events, a complication common to severe sepsis, have been associated with haptoglobin levels below the detection limit of typical measurements in burn patients [39]. Hence, this would seem to suggest that the dip in HP protein observed, could be a useful new biomarker for sepsis progression.

Moreover, free hemoglobin and haptoglobin were found to change in conjunction in some patients of the surviving group, whereas this kind of link was not seen in the adverse outcome group. In addition, the adverse outcome group exhibited less of an overall increase in haptoglobin than the surviving group. These results were supported by the subsequent Western blot of haptoglobin (Fig 11). A potential explanation for these results could be that haptoglobin binding of free heme was more effective in the surviving group.

Moreover, in some patients in the surviving group, free hemoglobin and haptoglobin were observed to change in conjunction, whereas this kind of link was not seen in the poor outcome group in this study. The poor outcome group also exhibited less of an increase in haptoglobin than the surviving group. Altogether, these results suggest that in critically ill sepsis patients, the production of haptoglobin cannot be induced as normal, enabling sepsis to progress in severity.

Finally, a sudden marked decrease in ceruloplasmin was observed that could be indicative of imminent liver failure, a complication often observed in sepsis. Although drops in plasma levels are generally associated with liver malfunction, ceruloplasmin has not been linked specifically to sepsis progression prior to this study.

In previous studies, serum proteomics by isobaric labeling have implied ceruloplasmin as a potential marker for graft-versus-host disease [40]. We now present that it may also be associated with sepsis progression.

The spike-and-dip approach implemented in the current study has two practical advantages. While it is tempting to screen for any kind of non-linear change, this approach is beneficial because (1) The number of required comparisons is reduced by considering only spots where a certain change, followed by a change in the opposite direction is observed; (2) Sudden dips or spikes in levels of proteins are easy to detect and act upon in a clinical setting, allowing for more timely diagnosis of alarming progression of the condition.

The identification by tandem mass spectrometry revealed that these dips could very well be linked to the organ dysfunction often observed in severe sepsis cases. Nonetheless, it is important to note that of the 83 spots compared in the current study, only 49 could be successfully identified. Thus, it is possible that additional biomarkers can be suggested upon further analysis of the remaining 34 spots.

In addition, our study also emphasizes the usefulness of partial correlations as opposed to the more popular heatmaps of marginal correlations. Identification yielded clusters of tightly connected spots corresponding to known proteins, or proteins with known shared biological functions. Contrary to heatmaps, the resulting sparse graphs show only direct relationships between variables and are far less cluttered with spurious correlations. A major advantage of proteomics over the study of individual proteins with respect to this approach, is that the construction of these networks does not depend on the identification of proteins belonging to spots. This means that unknown spots can be modelled as well and helps counter the effect of potential confounders.

In conclusion, our research demonstrates how longitudinal proteomics data can be used to screen for biomarkers en masse, superseding the search for individual proteins involved in complex conditions like sepsis. Both for the discovery of biomarkers and for their application in a clinical setting, the two-dimensional gel electrophoresis (2D-PAGE) approach utilized in this study can include a multitude of relevant proteins simultaneously. Future studies with larger sample sizes can continue to explore this technique and validate these results in terms of predictive performance of sepsis progression.

## Supporting information

S1 Fig Two-dimensional electrophoresis images of the serum of Patient 2. A) First day, B) second day, C) third day, D) fifth day, and E) seventh day.
(TIF)

S2 Fig Two-dimensional electrophoresis images of the serum of Patient 3. A) First day, B) second day, C) third day, D) fifth day, and E) seventh day.
(TIF)

S3 Fig Two-dimensional electrophoresis images of the serum of Patient 5. A) First day, B) second day, C) third day, D) fifth day, and E) seventh day.
(TIF)

S4 Fig Two-dimensional electrophoresis images of the serum of Patient 8. A) First day, B) second day, C) third day, D) fifth day, and E) seventh day.
(TIF)

S5 Fig Two-dimensional electrophoresis images of the serum of Patient 9. A) First day, B) second day, C) third day, D) fifth day, and E) seventh day.
(TIF)

S6 Fig Two-dimensional electrophoresis images of the serum of Patient 17. A) First day, B) second day, C) third day, D) fifth day, and E) seventh day.
(TIF)

S7 Fig Two-dimensional electrophoresis images of the serum of Patient 18. A) First day, B) second day, C) third day, D) fifth day, and E) seventh day.
(TIF)

S8 Fig Two-dimensional electrophoresis images of the serum of Patient 19. A) First day, B) second day, C) third day, D) fifth day, and E) seventh day.
(TIF)

S9 Fig Two-dimensional electrophoresis images of the serum of Patient 20. A) First day, B) second day, C) third day, D) fifth day, and E) seventh day.
(TIF)

S10 Fig Two-dimensional electrophoresis images of the serum of Patient 21. A) First day, B) second day, C) third day, D) fifth day, and E) seventh day.
(TIF)

S11 Fig Two-dimensional electrophoresis images of the serum of Patient 22. A) First day, B) second day, C) third day, D) fifth day, and E) seventh day.
(TIF)

S12 Fig Two-dimensional electrophoresis images of the serum of Patient 23. A) First day, B) second day, C) third day, D) fifth day, and E) seventh day.
(TIF)

S13 Fig Two-dimensional electrophoresis images of the serum of Patient 24. A) First day, B) second day, C) third day, D) fifth day, and E) seventh day.
(TIF)

S14 Fig Two-dimensional electrophoresis images of the serum of Patient 25. A) First day, B) second day, C) third day, D) fifth day, and E) seventh day.
(TIF)

S15 Fig Two-dimensional electrophoresis images of the serum of Patient 27. A) First day, B) second day, C) third day, D) fifth day, and E) seventh day.
(TIF)

S16 Fig Two-dimensional electrophoresis images of the serum of Patient 28. A) First day, B) second day, C) third day, D) fifth day, and E) seventh day.
(TIF)

S17 Fig Two-dimensional electrophoresis images of the serum of Patient 29. A) First day, B) second day, C) third day, D) fifth day, and E) seventh day.
(TIF)

S18 Fig Two-dimensional electrophoresis images of the serum of Patient 30. A) First day, B) second day, C) third day, D) fifth day, and E) seventh day.
(TIF)

S19 Fig Two-dimensional electrophoresis images of the serum of Patient 31. A) First day, B) second day, C) third day, D) fifth day, and E) seventh day.
(TIF)

S20 Fig Heatmaps of marginal correlations of time point transitions *t*_2_→*t*_3_. Row and column order was set by hierarchical clustering using 1−*cor*(*x*), or 1−*pcor*(*x*) as distance function for the marginal and partial correlations, respectively. Color represents positive (red) or negative (blue) correlations.
(TIFF)

S21 Fig Heatmaps of marginal correlations of time point transitions *t*_3_→*t*_4_. Row and column order was set by hierarchical clustering using 1−*cor*(*x*), or 1−*pcor*(*x*) as distance function for the marginal and partial correlations, respectively. Color represents positive (red) or negative (blue) correlations.
(TIFF)

S22 Fig Heatmaps of marginal correlations of time point transitions *t*_4_→*t*_5_. Row and column order was set by hierarchical clustering using 1−*cor*(*x*), or 1−*pcor*(*x*) as distance function for the marginal and partial correlations, respectively. Color represents positive (red) or negative (blue) correlations.
(TIFF)

S23 Fig Heatmaps of partial correlations of time point transitions *t*_2_→*t*_3_. Row and column order was set by hierarchical clustering using 1−*cor*(*x*), or 1−*pcor*(*x*) as distance function for the marginal and partial correlations, respectively. Color represents positive (red) or negative (blue) correlations.
(TIFF)

S24 Fig Heatmaps of partial correlations of time point transitions *t*_3_→*t*_4_. Row and column order was set by hierarchical clustering using 1−*cor*(*x*), or 1−*pcor*(*x*) as distance function for the marginal and partial correlations, respectively. Color represents positive (red) or negative (blue) correlations.
(TIFF)

S25 Fig Heatmaps of partial correlations of time point transitions *t*_4_→*t*_5_. Row and column order was set by hierarchical clustering using 1−*cor*(*x*), or 1−*pcor*(*x*) as distance function for the marginal and partial correlations, respectively. Color represents positive (red) or negative (blue) correlations.
(TIFF)

## Abbreviations

2D-PAGE: Two-dimensional gel electrophoresis / Two-dimensional polyacrylamide gel electrophoresis
HP-2D-PAGE: High-performance two-dimensional polyacrylamide gel electrophoresis
GGM: Gaussian graphical model
SOFA: Sequential organ failure assessment
qSOFA: Quick sequential organ failure assessment
IL-6: Interleukin-6
PCT: Procalcitonin
SAPS II: Simplified acute physiology score II
CAN: acetonitrile
DTT: dithiothreitol
TFA: trifluoroacetic acid
LC/MS/MS: liquid chromatography-tandem mass spectrometry
Q-TOF: Quadrupole-time-of-flight
PLGS: ProteinLynx Global Server
LOOCV: Leave one out cross validation
HP: haptoglobin
TTR: Transythyretin
CRP: C-reactive protein
vWF: von Willebrand factor
ADAMTS13: a thrombospondin type I motif, member 13
