# Supplementary figures and images for: Multiple biomarkers of sepsis identified by novel time-lapse proteomics of patient serum
Source: PLoS One. 2019 Sep 30;14(9):e0222403. doi: 10.1371/journal.pone.0222403 (PMC6768476; doi:10.1371/journal.pone.0222403)

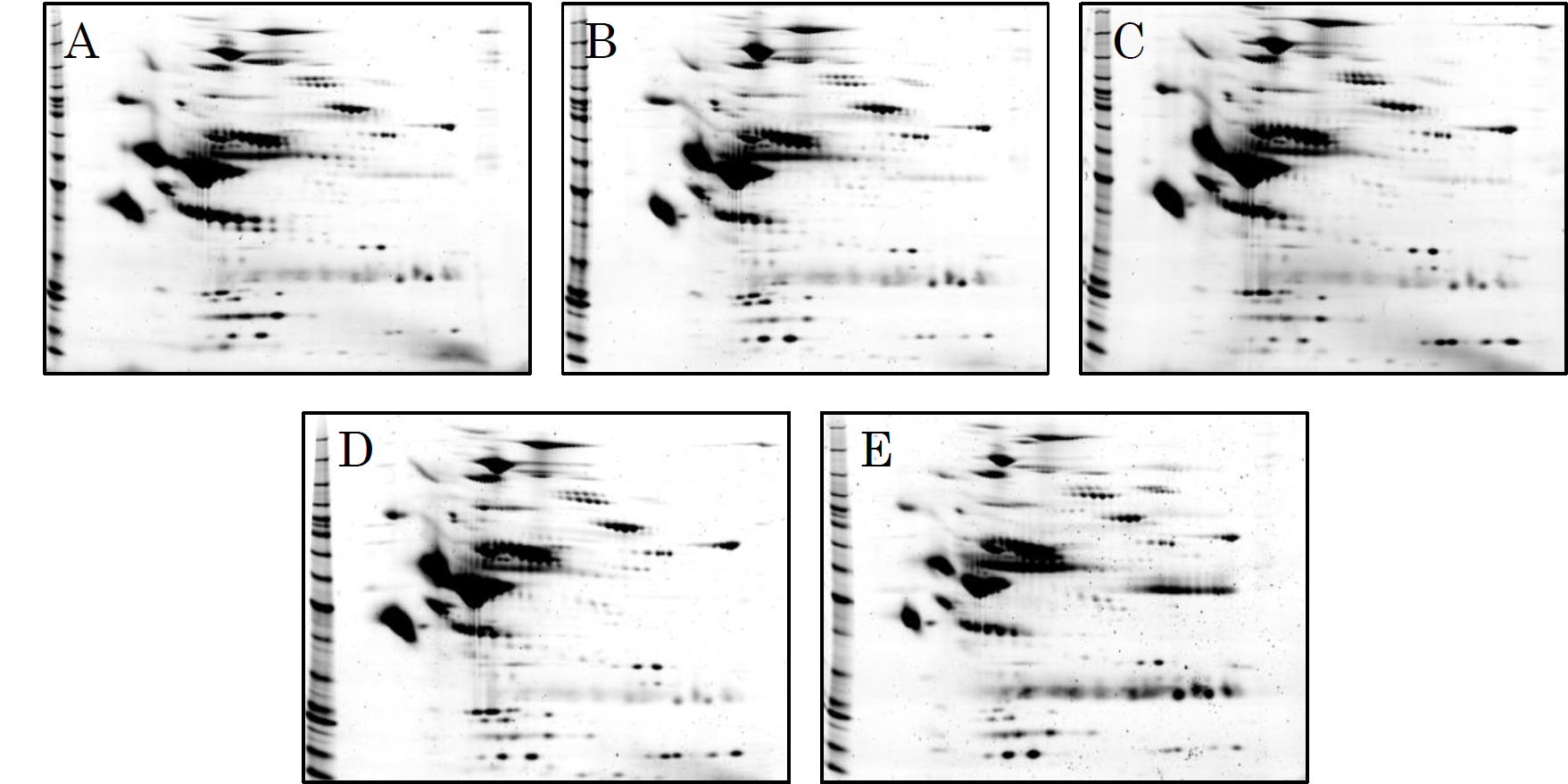

Supplement: S1 Fig — A) First day, B) second day, C) third day, D) fifth day, and E) seventh day. (TIF) [file pone.0222403.s001.tif]

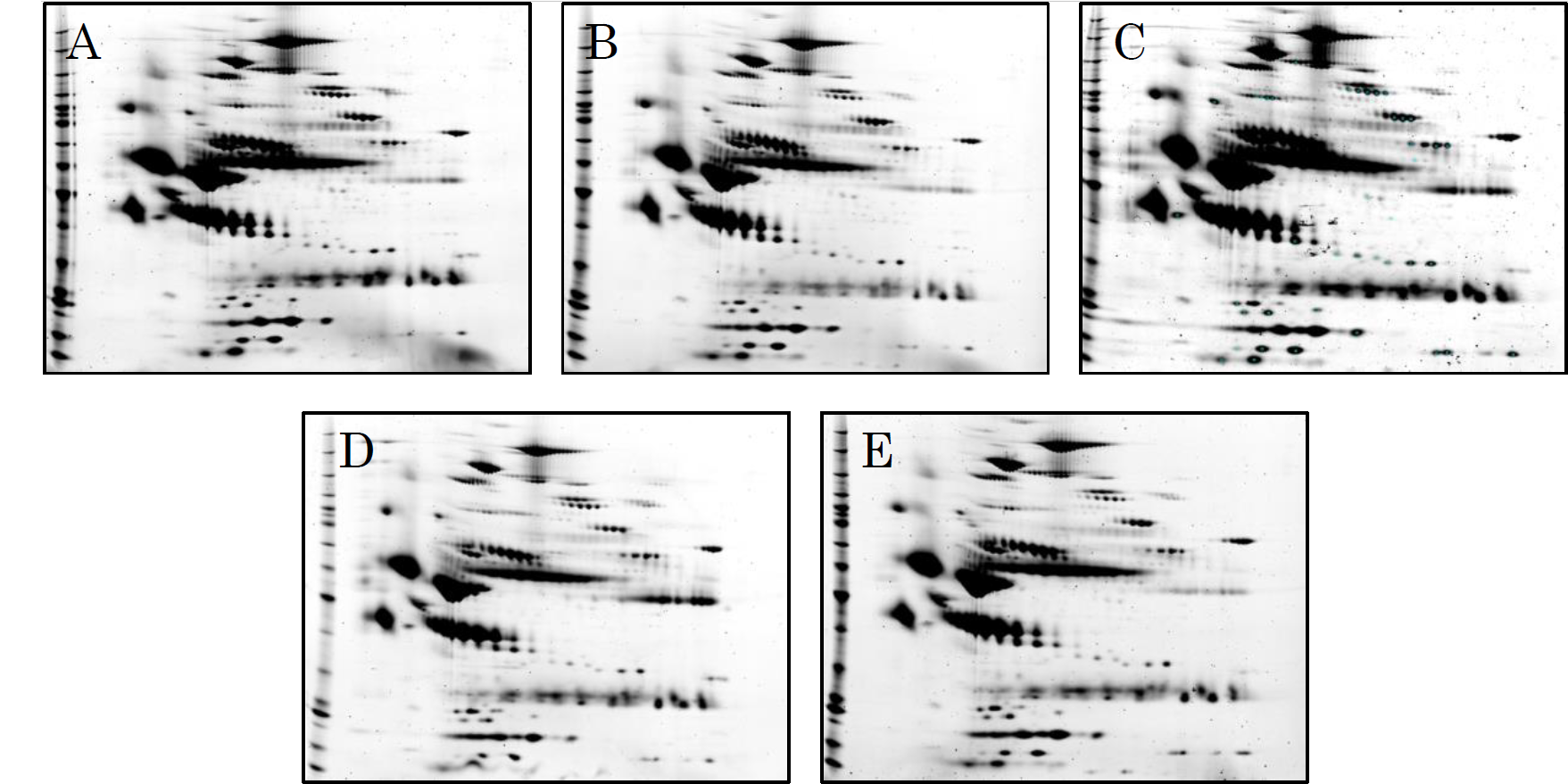

Supplement: S2 Fig — A) First day, B) second day, C) third day, D) fifth day, and E) seventh day. (TIF) [file pone.0222403.s002.tif]

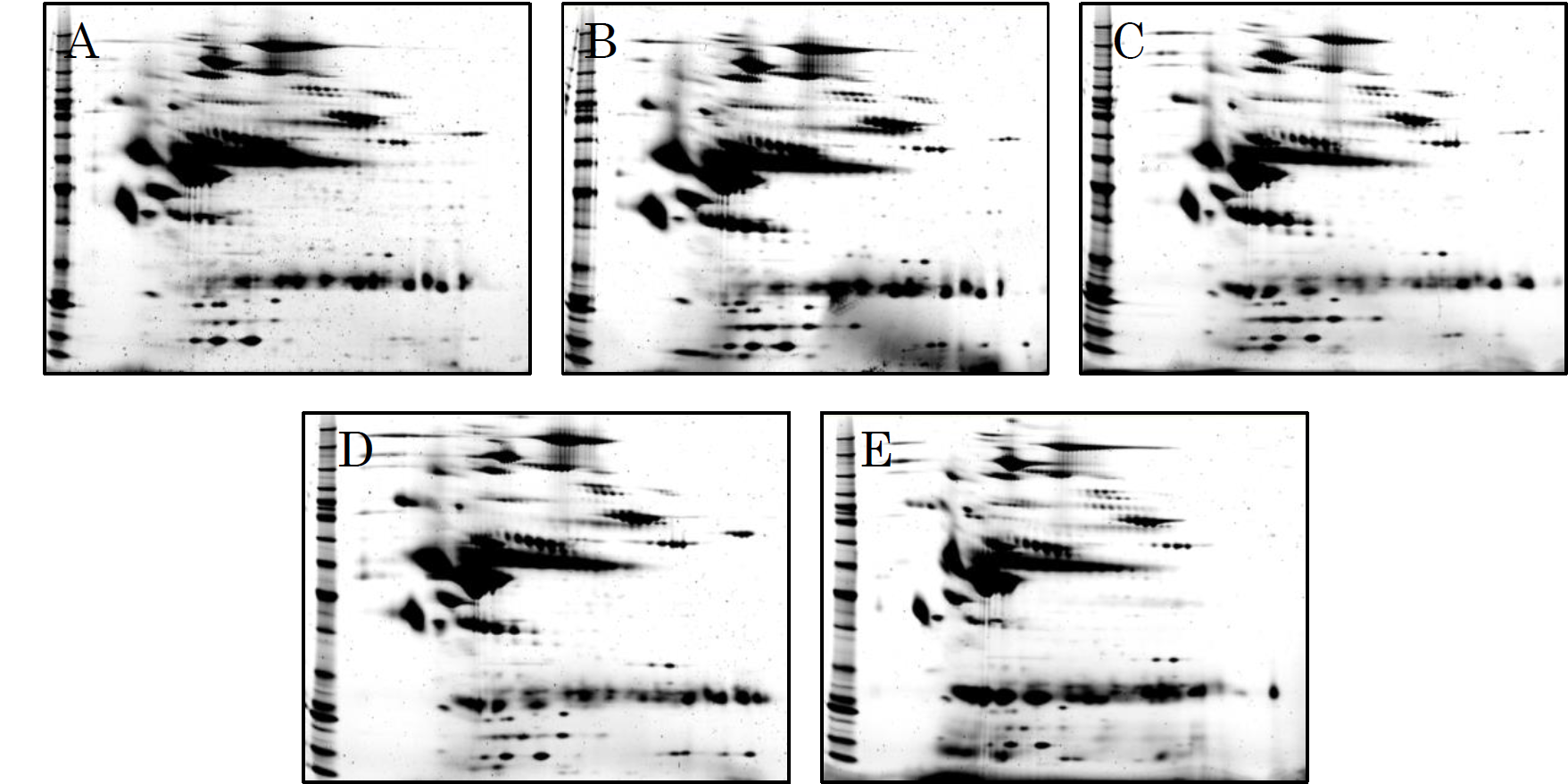

Supplement: S3 Fig — A) First day, B) second day, C) third day, D) fifth day, and E) seventh day. (TIF) [file pone.0222403.s003.tif]

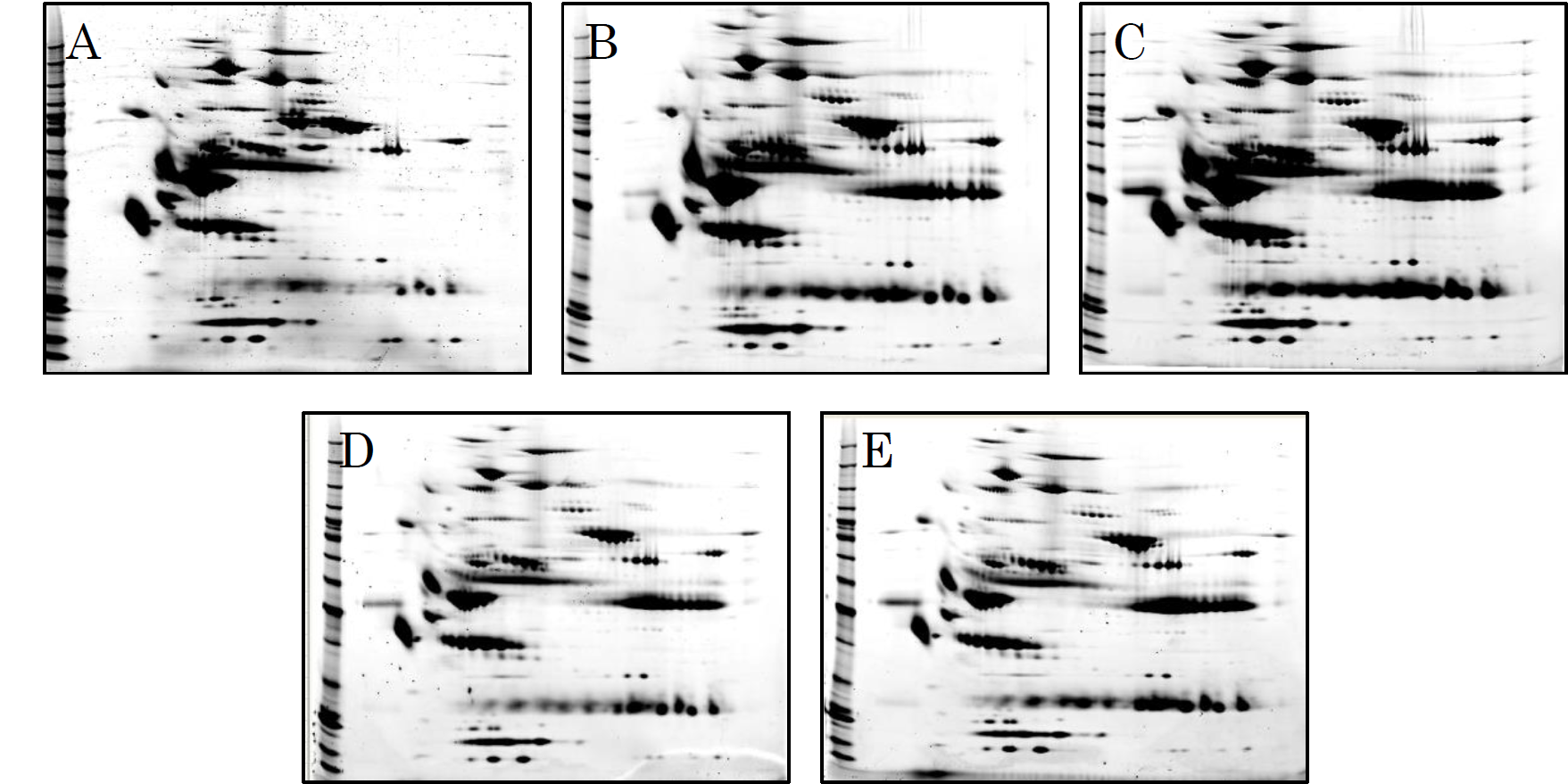

Supplement: S4 Fig — A) First day, B) second day, C) third day, D) fifth day, and E) seventh day. (TIF) [file pone.0222403.s004.tif]

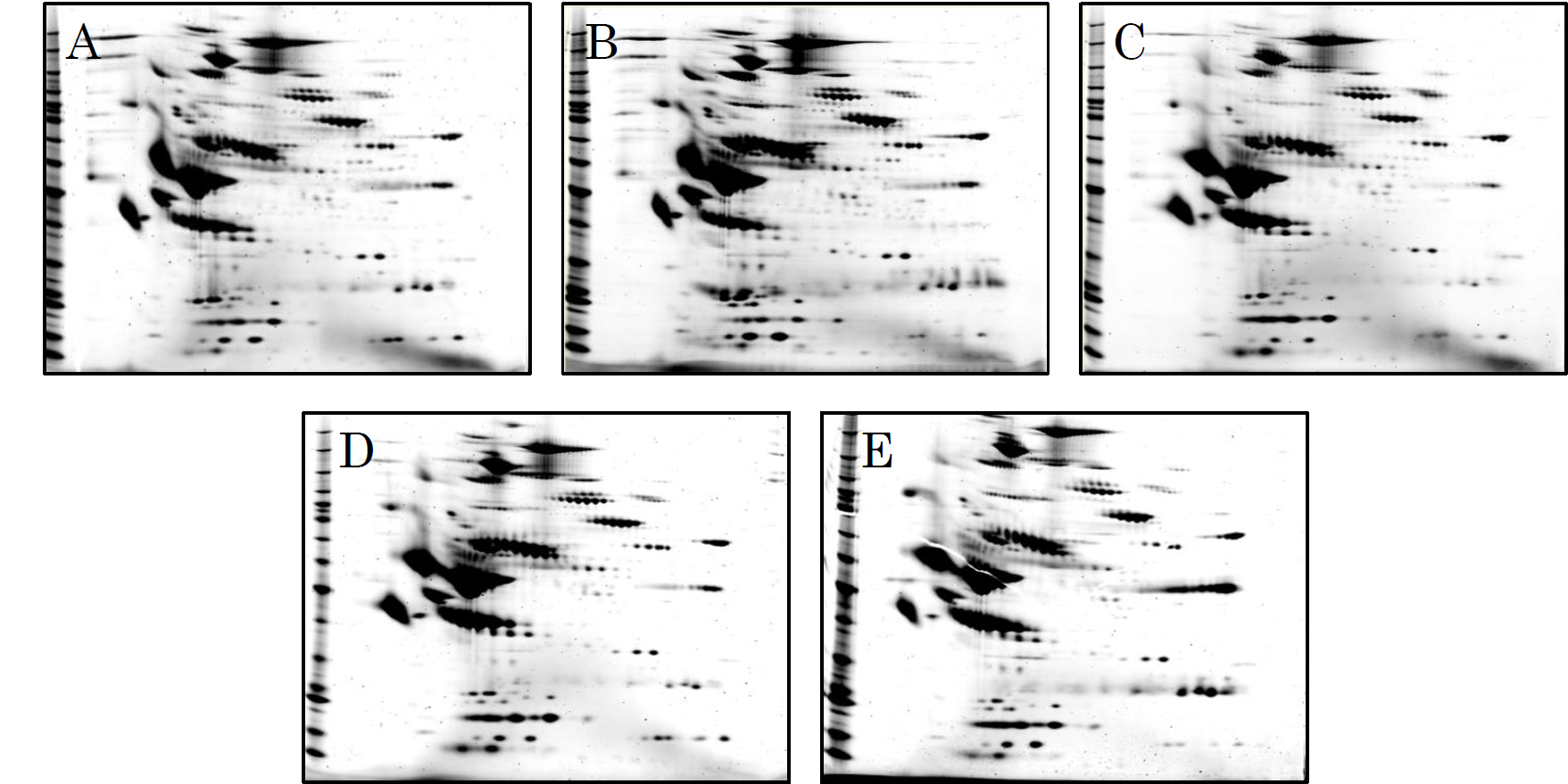

Supplement: S5 Fig — A) First day, B) second day, C) third day, D) fifth day, and E) seventh day. (TIF) [file pone.0222403.s005.tif]

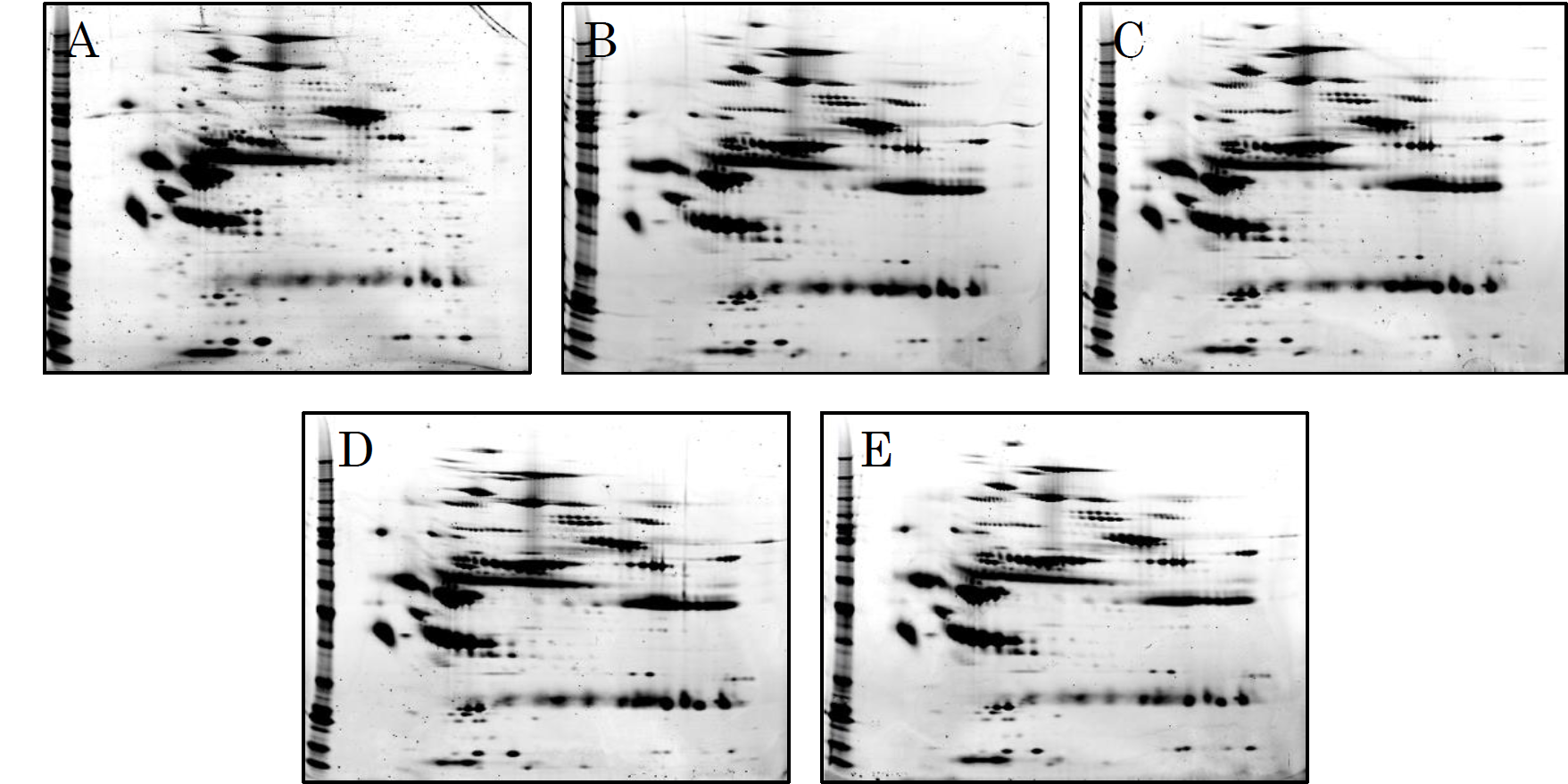

Supplement: S6 Fig — A) First day, B) second day, C) third day, D) fifth day, and E) seventh day. (TIF) [file pone.0222403.s006.tif]

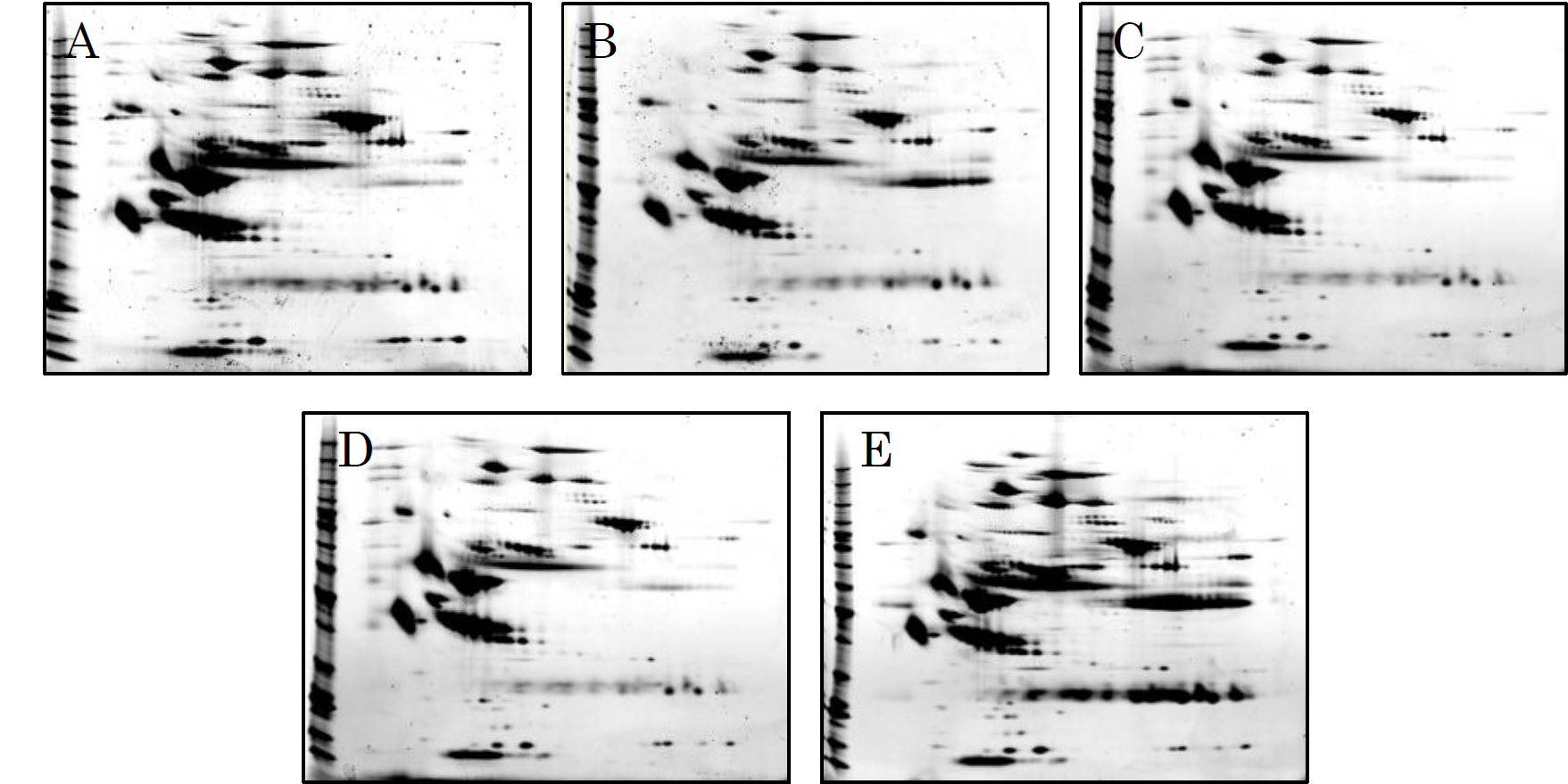

Supplement: S7 Fig — A) First day, B) second day, C) third day, D) fifth day, and E) seventh day. (TIF) [file pone.0222403.s007.tif]

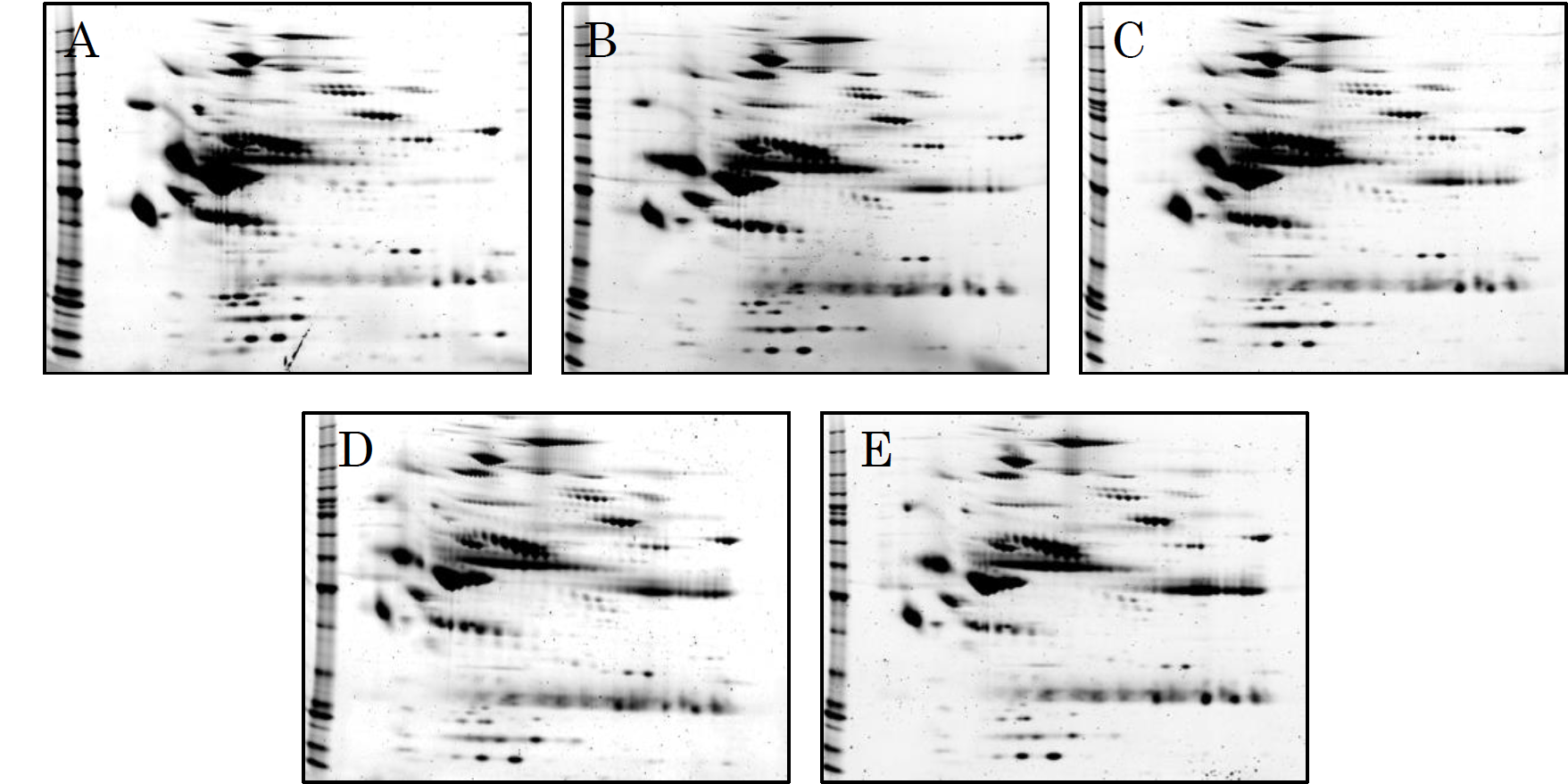

Supplement: S8 Fig — A) First day, B) second day, C) third day, D) fifth day, and E) seventh day. (TIF) [file pone.0222403.s008.tif]

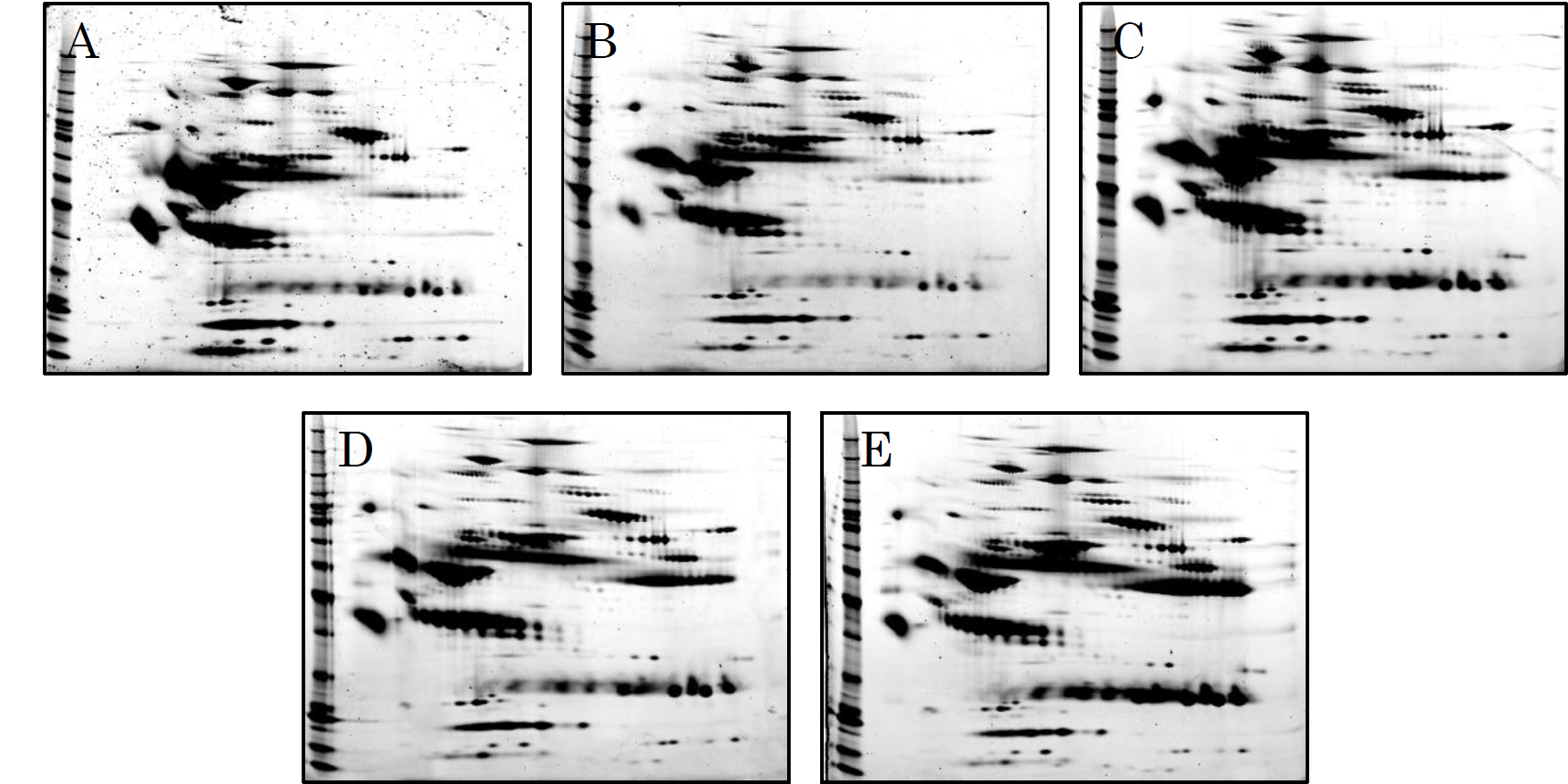

Supplement: S9 Fig — A) First day, B) second day, C) third day, D) fifth day, and E) seventh day. (TIF) [file pone.0222403.s009.tif]

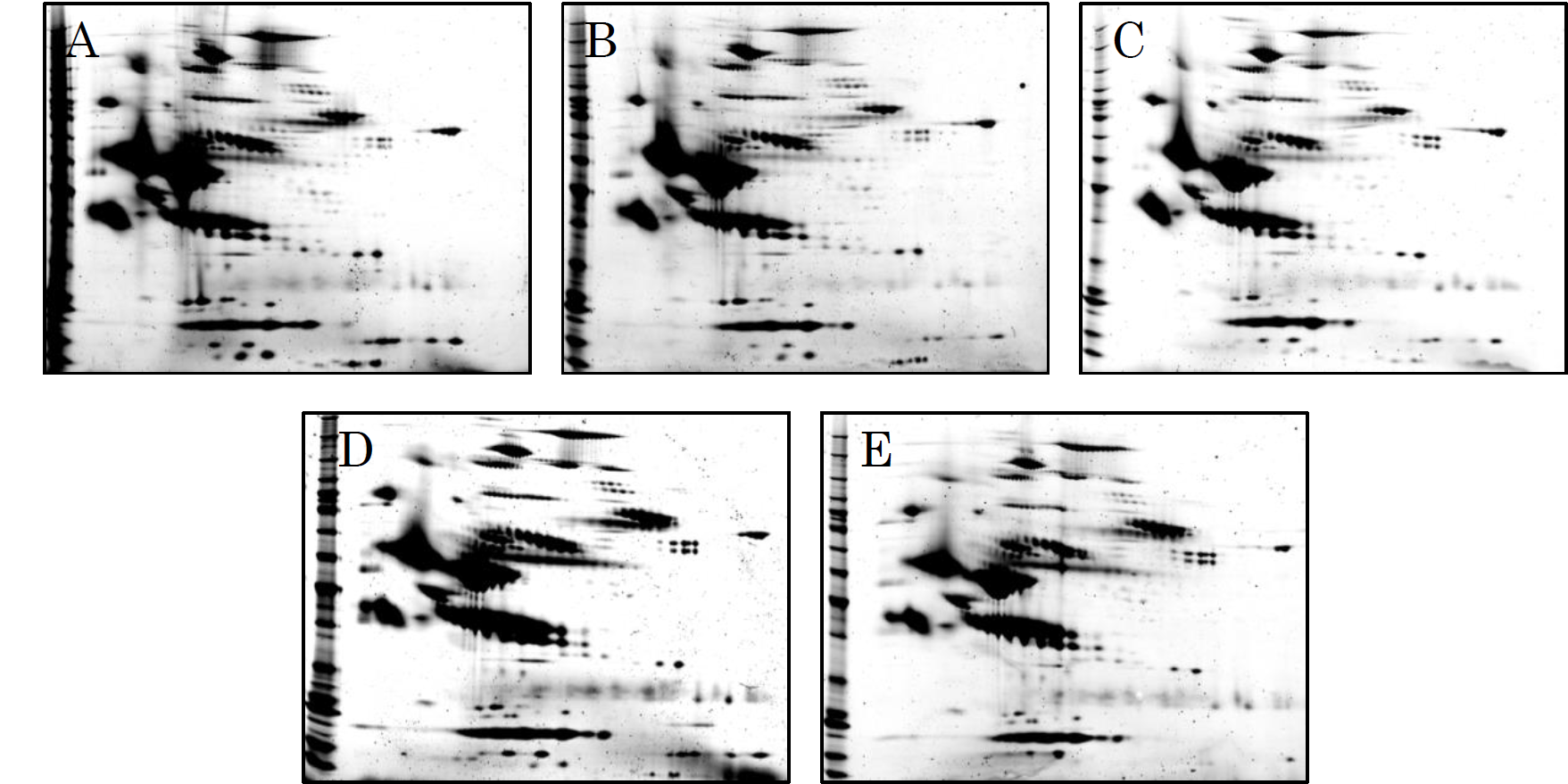

Supplement: S10 Fig — A) First day, B) second day, C) third day, D) fifth day, and E) seventh day. (TIF) [file pone.0222403.s010.tif]

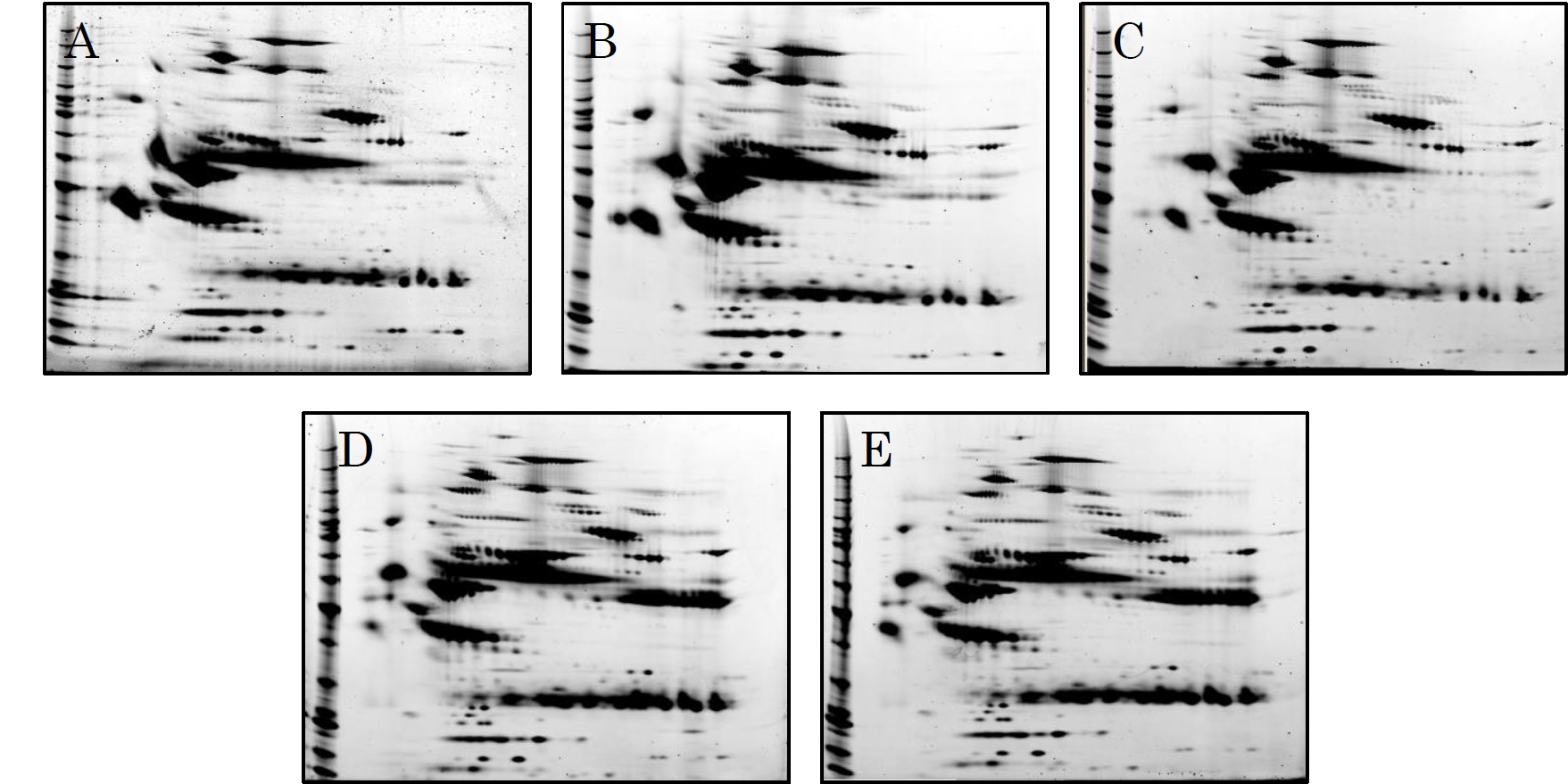

Supplement: S11 Fig — A) First day, B) second day, C) third day, D) fifth day, and E) seventh day. (TIF) [file pone.0222403.s011.tif]

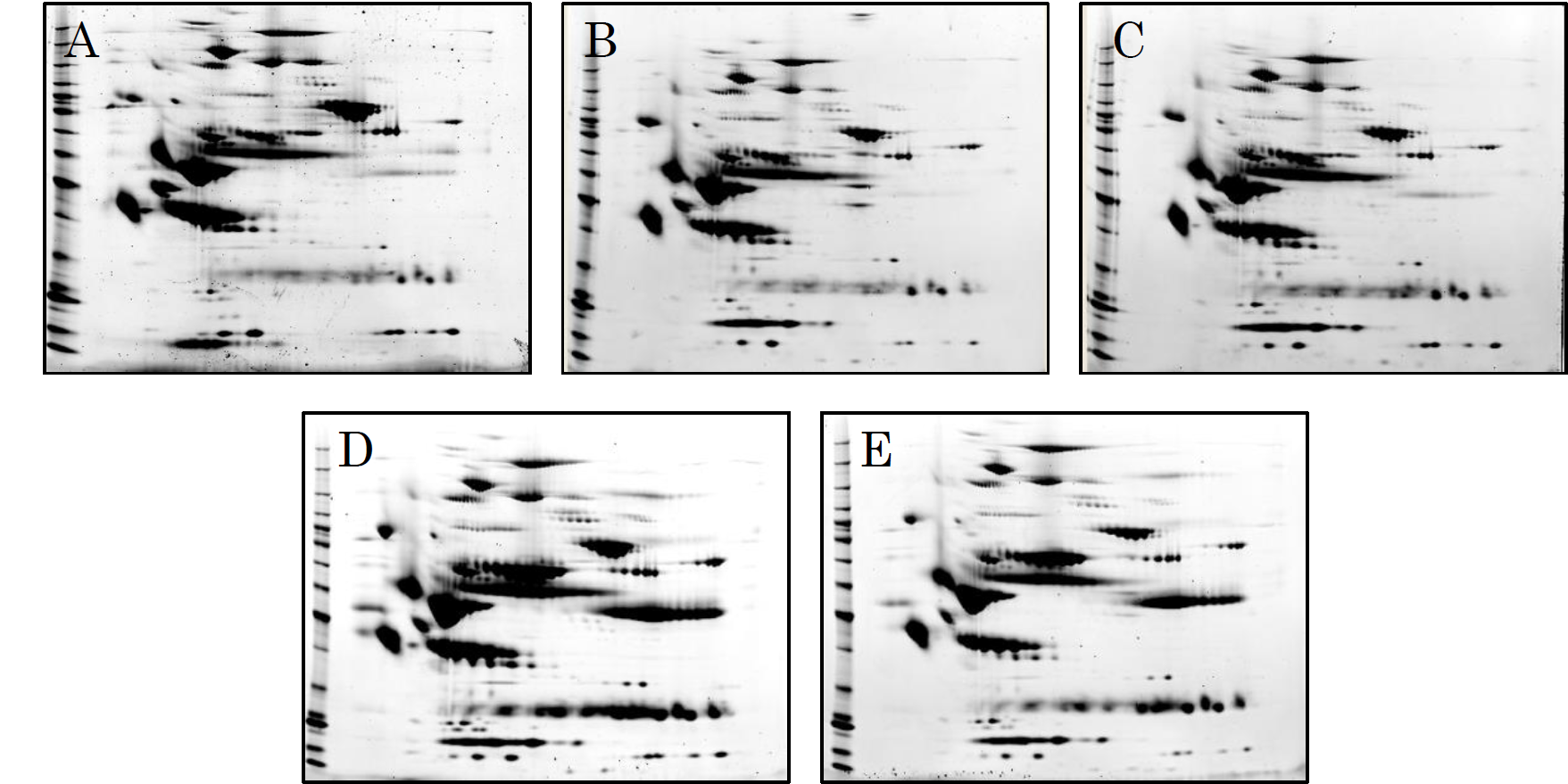

Supplement: S12 Fig — A) First day, B) second day, C) third day, D) fifth day, and E) seventh day. (TIF) [file pone.0222403.s012.tif]

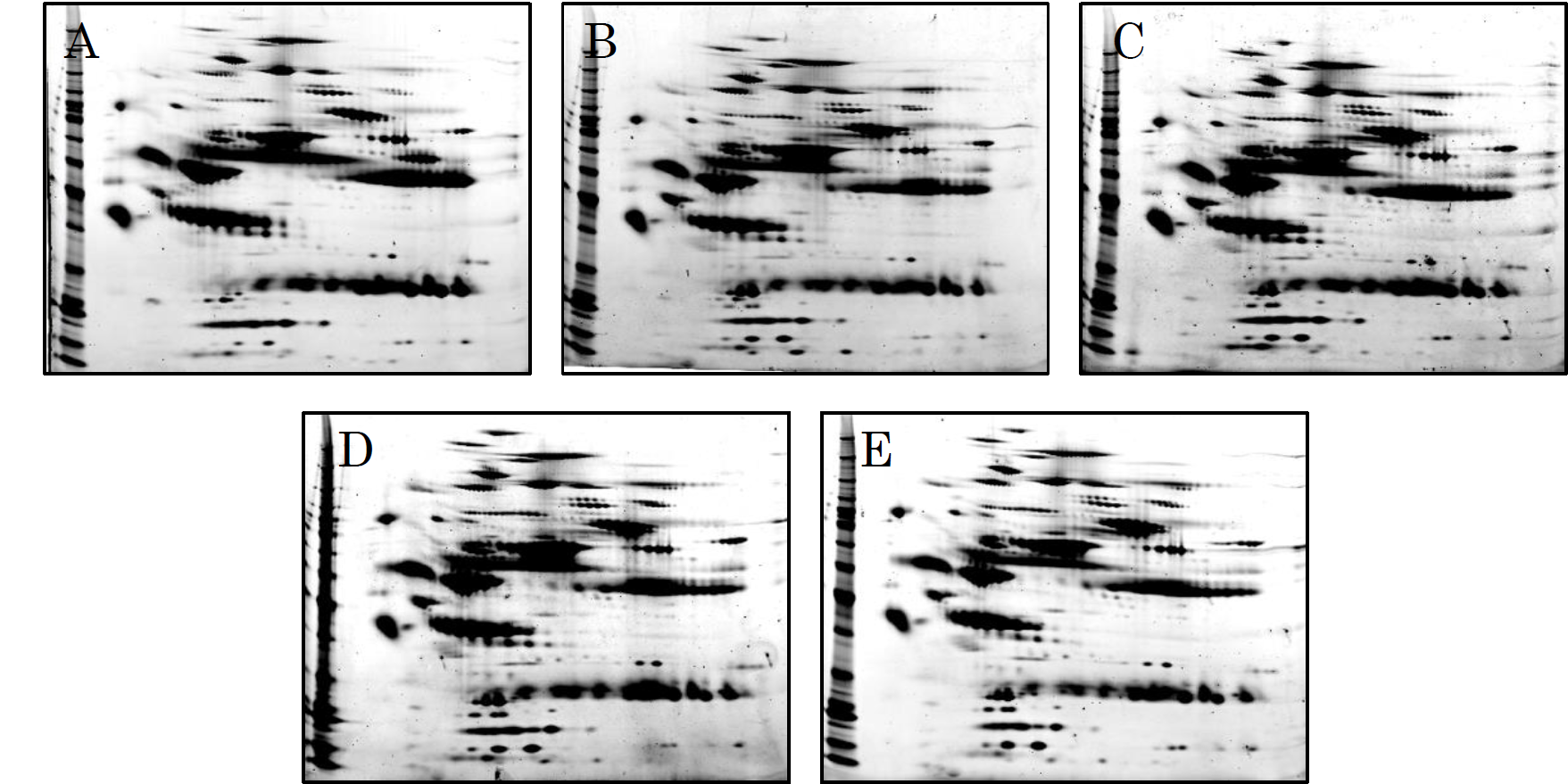

Supplement: S13 Fig — A) First day, B) second day, C) third day, D) fifth day, and E) seventh day. (TIF) [file pone.0222403.s013.tif]

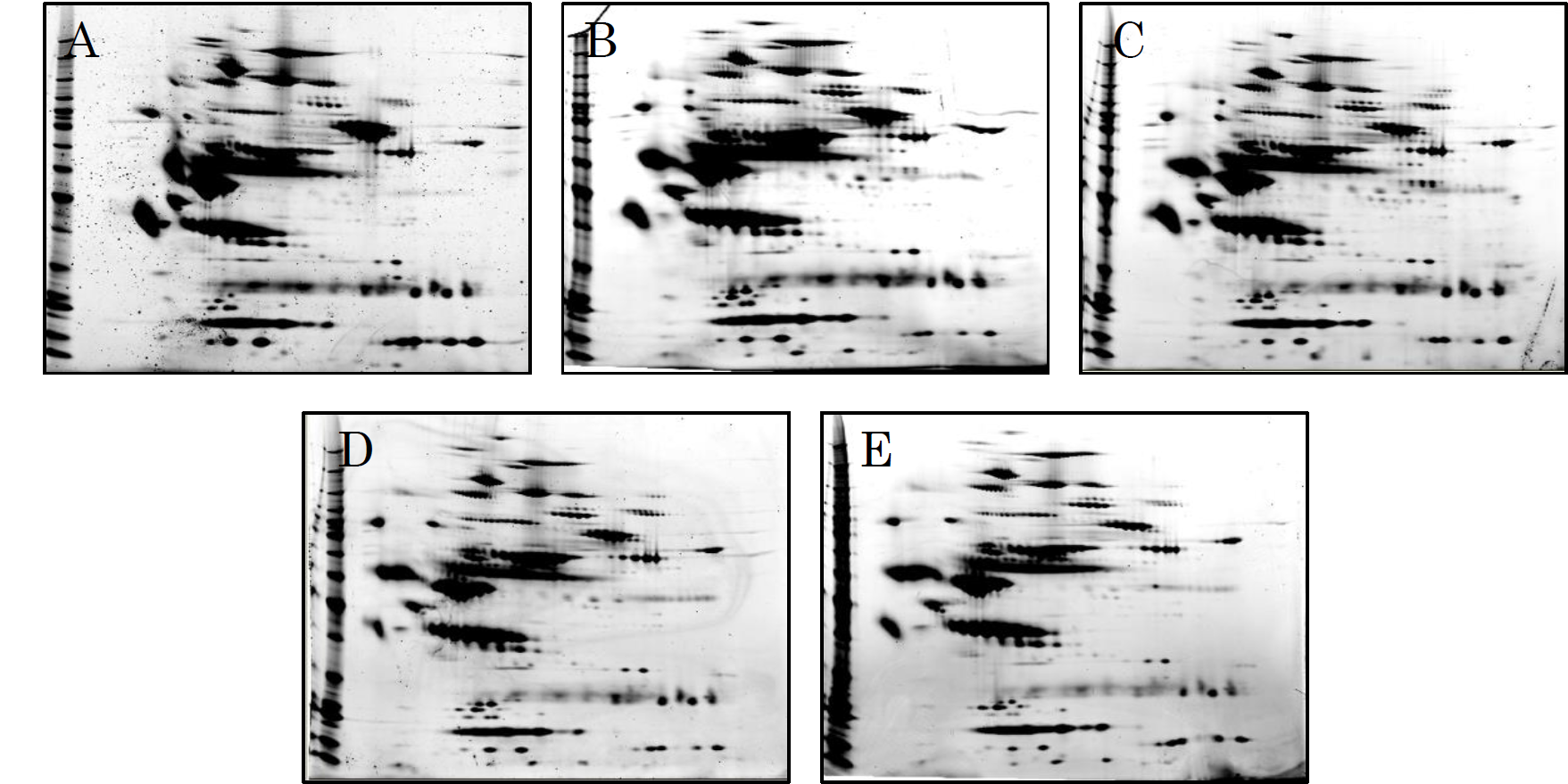

Supplement: S14 Fig — A) First day, B) second day, C) third day, D) fifth day, and E) seventh day. (TIF) [file pone.0222403.s014.tif]

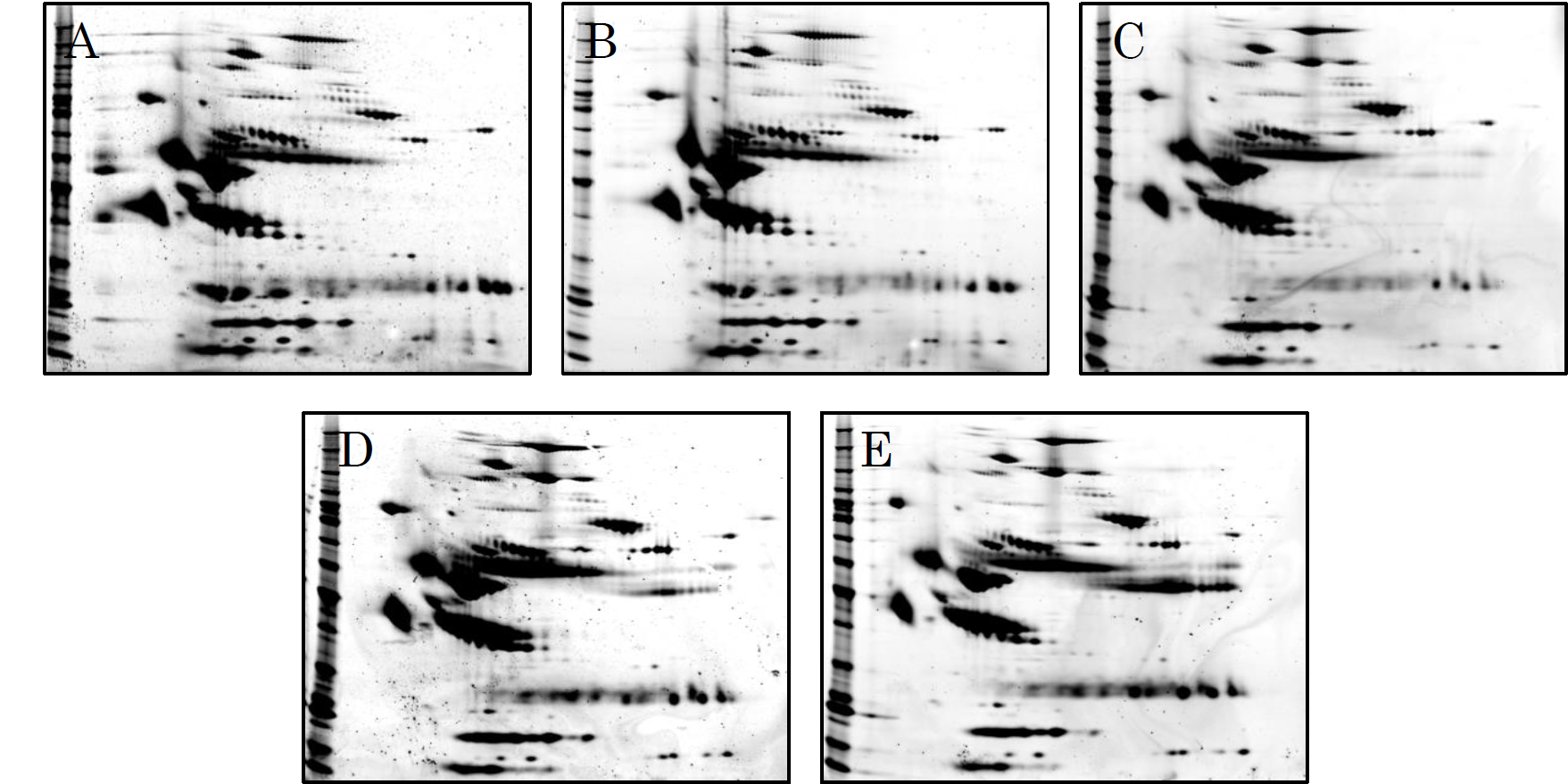

Supplement: S15 Fig — A) First day, B) second day, C) third day, D) fifth day, and E) seventh day. (TIF) [file pone.0222403.s015.tif]

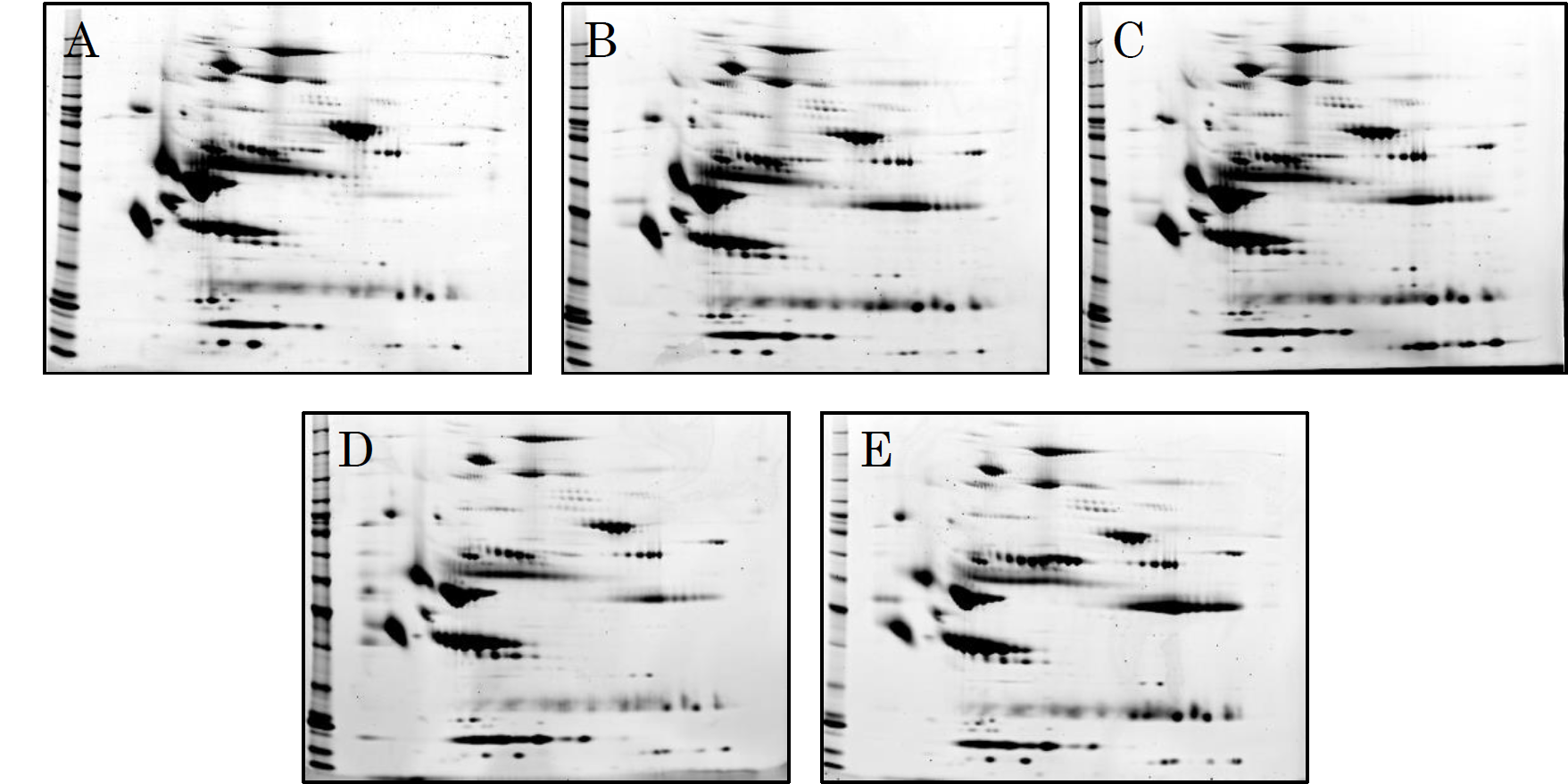

Supplement: S16 Fig — A) First day, B) second day, C) third day, D) fifth day, and E) seventh day. (TIF) [file pone.0222403.s016.tif]

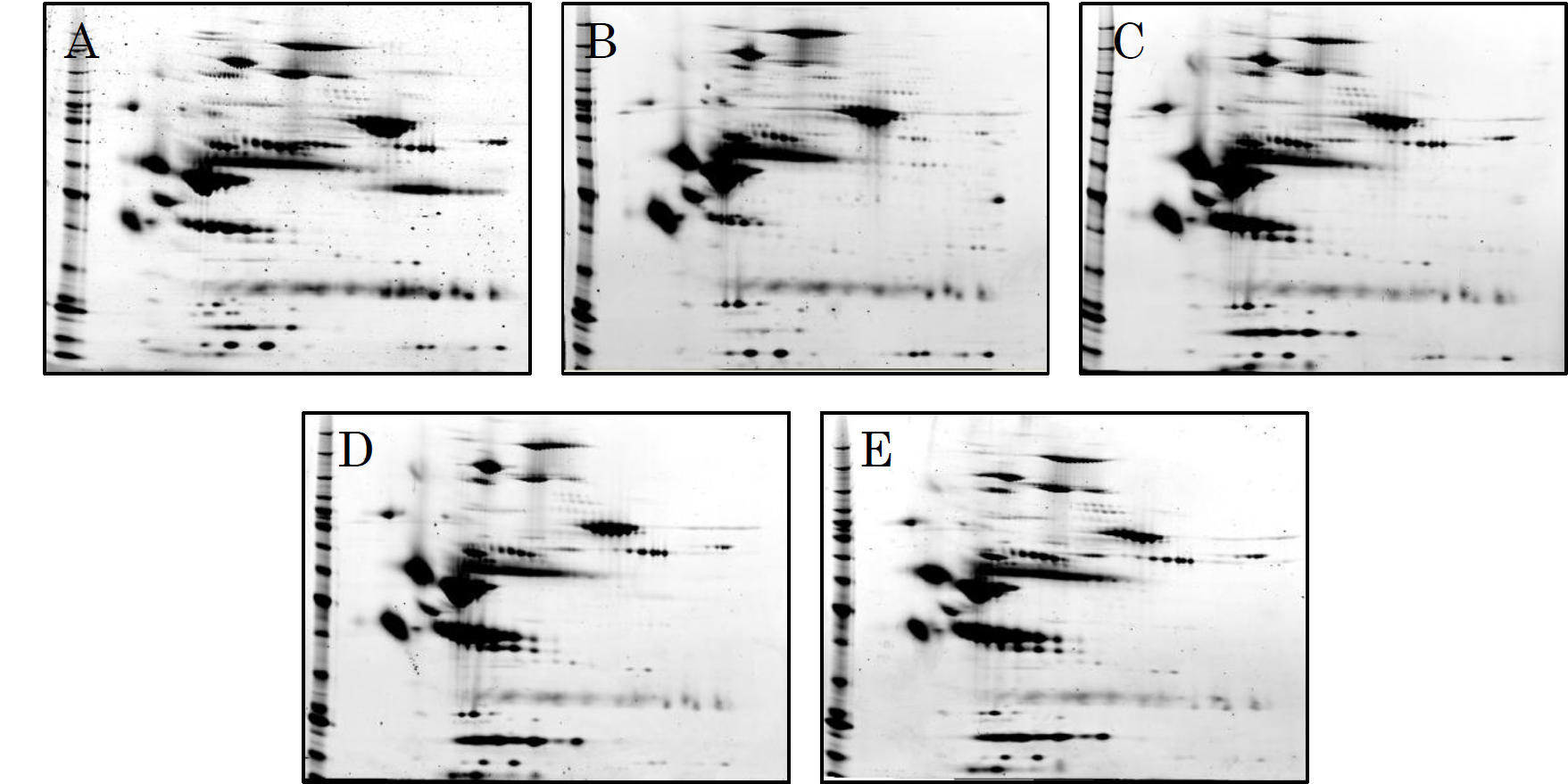

Supplement: S17 Fig — A) First day, B) second day, C) third day, D) fifth day, and E) seventh day. (TIF) [file pone.0222403.s017.tif]

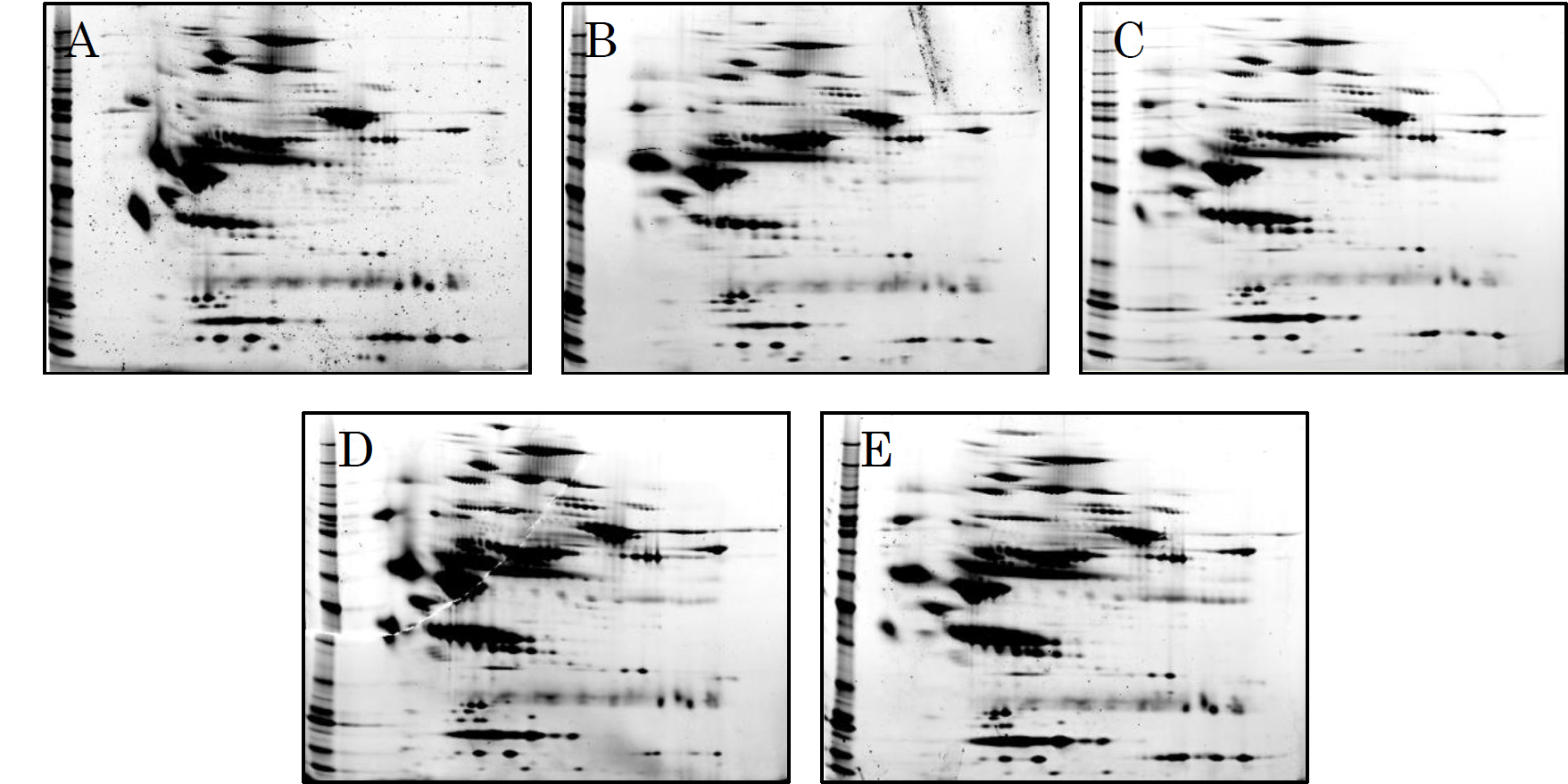

Supplement: S18 Fig — A) First day, B) second day, C) third day, D) fifth day, and E) seventh day. (TIF) [file pone.0222403.s018.tif]

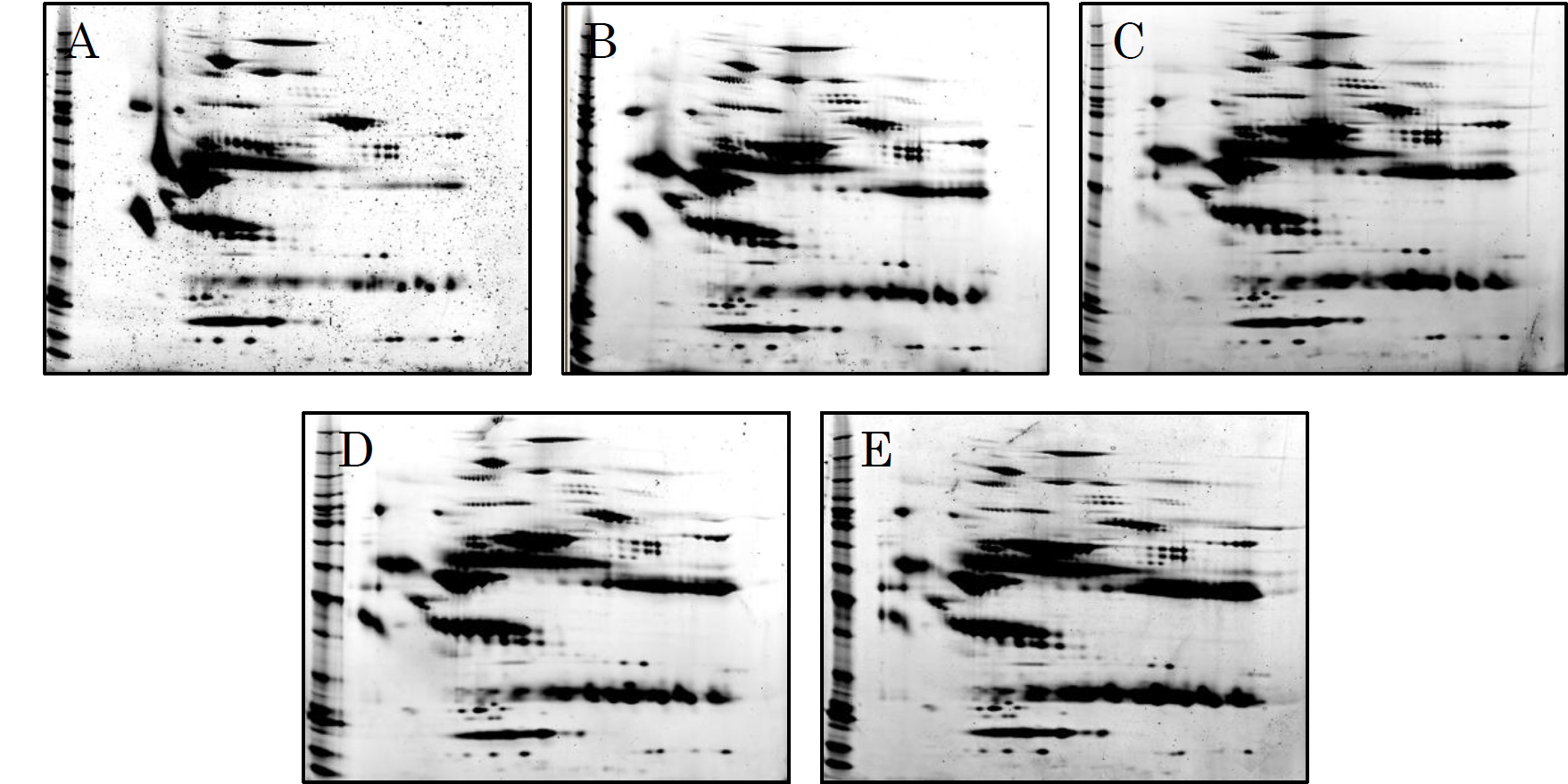

Supplement: S19 Fig — A) First day, B) second day, C) third day, D) fifth day, and E) seventh day. (TIF) [file pone.0222403.s019.tif]

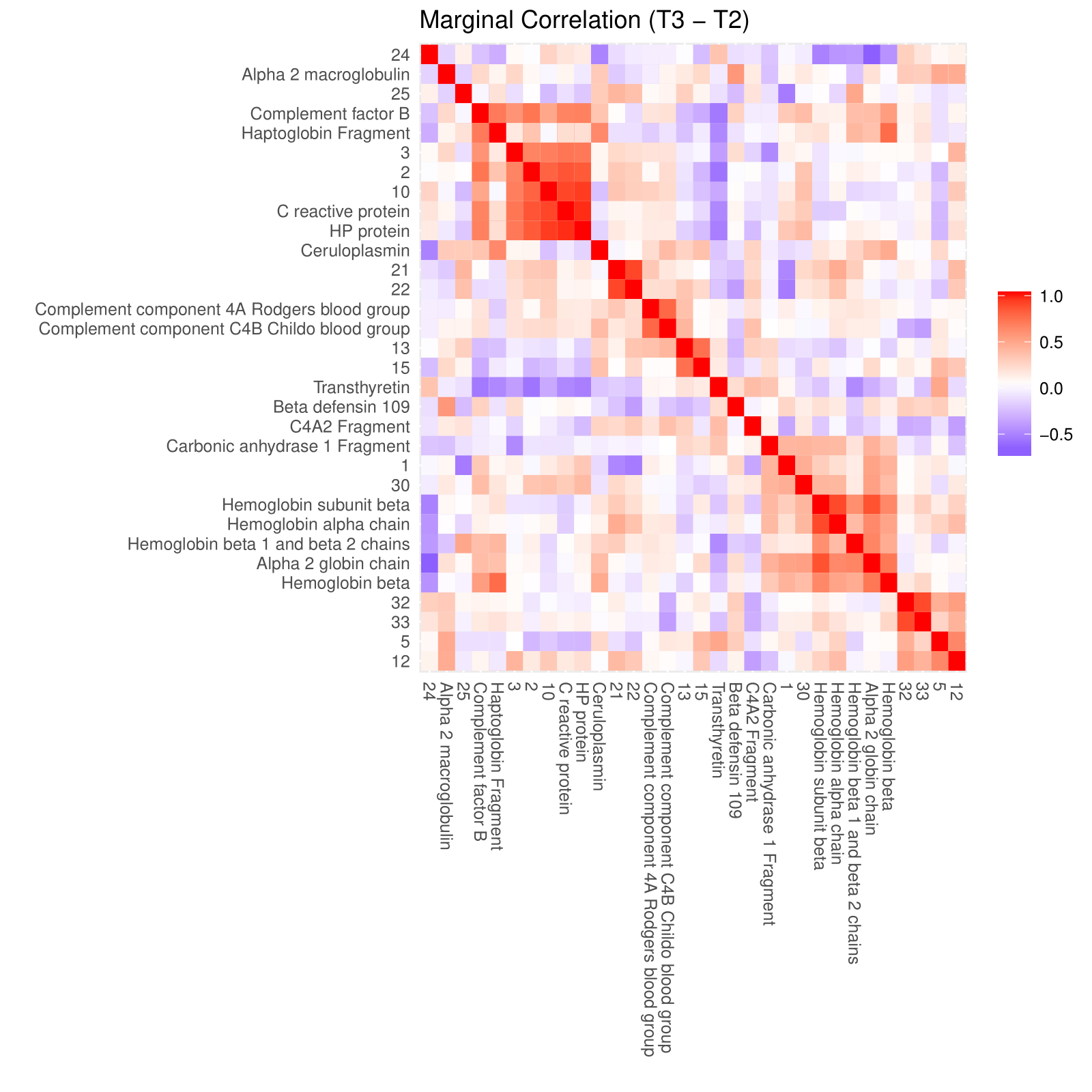

Supplement: S20 Fig — Row and column order was set by hierarchical clustering using 1−cor(x), or 1−pcor(x) as distance function for the marginal and partial correlations, respectively. Color represents positive (red) or negative (blue) correlations. (TIFF) [file pone.0222403.s020.tiff]

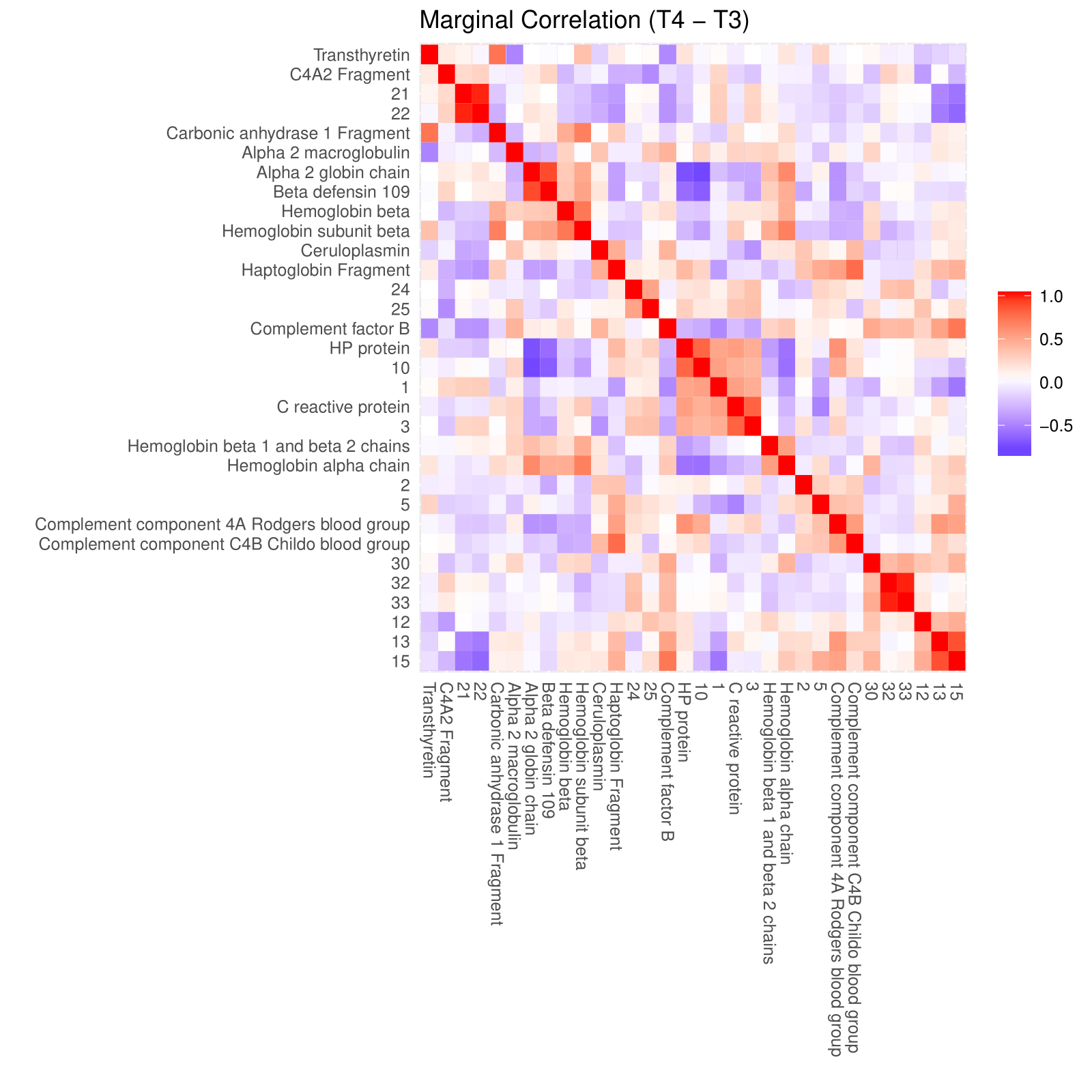

Supplement: S21 Fig — Row and column order was set by hierarchical clustering using 1−cor(x), or 1−pcor(x) as distance function for the marginal and partial correlations, respectively. Color represents positive (red) or negative (blue) correlations. (TIFF) [file pone.0222403.s021.tiff]

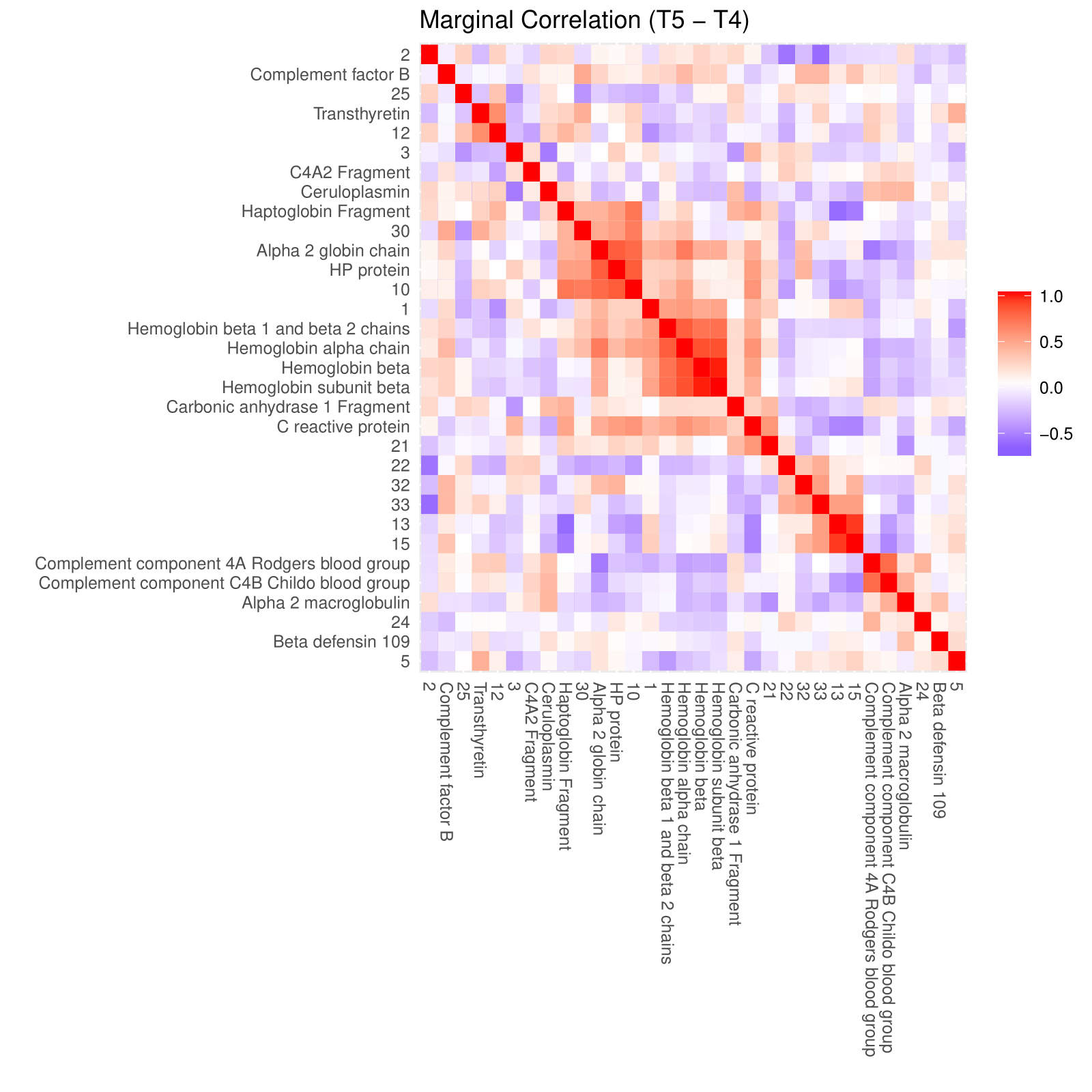

Supplement: S22 Fig — Row and column order was set by hierarchical clustering using 1−cor(x), or 1−pcor(x) as distance function for the marginal and partial correlations, respectively. Color represents positive (red) or negative (blue) correlations. (TIFF) [file pone.0222403.s022.tiff]

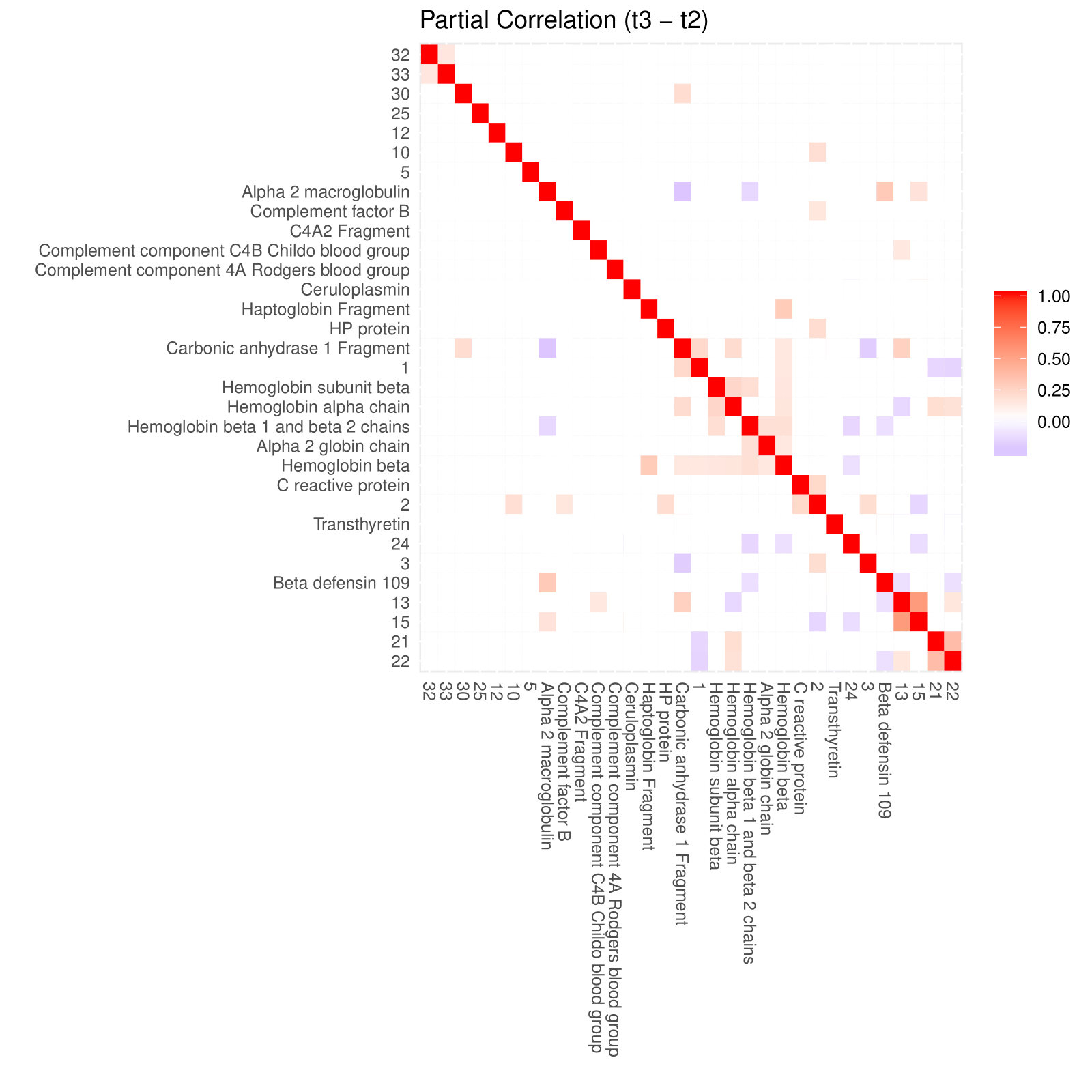

Supplement: S23 Fig — Row and column order was set by hierarchical clustering using 1−cor(x), or 1−pcor(x) as distance function for the marginal and partial correlations, respectively. Color represents positive (red) or negative (blue) correlations. (TIFF) [file pone.0222403.s023.tiff]

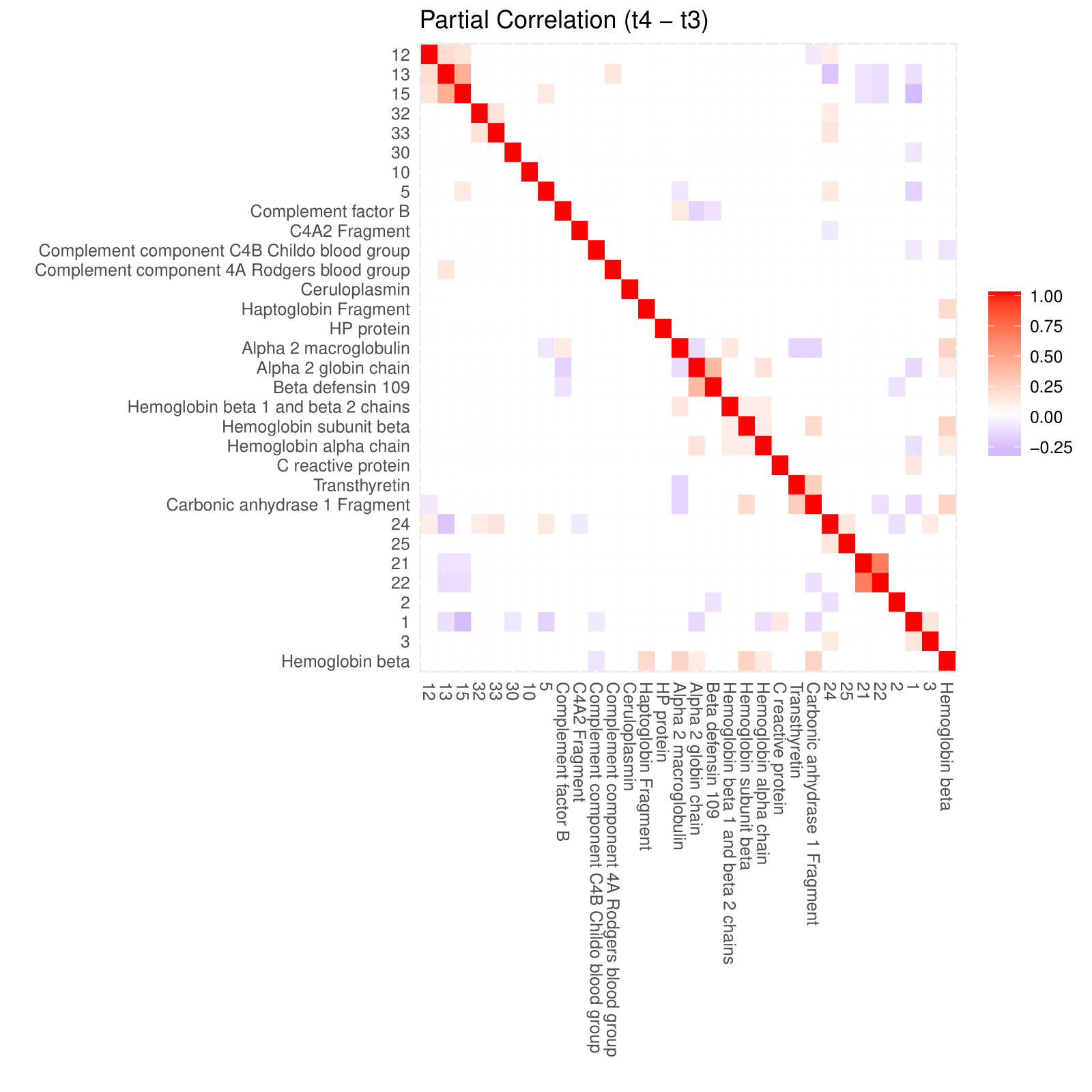

Supplement: S24 Fig — Row and column order was set by hierarchical clustering using 1−cor(x), or 1−pcor(x) as distance function for the marginal and partial correlations, respectively. Color represents positive (red) or negative (blue) correlations. (TIFF) [file pone.0222403.s024.tiff]

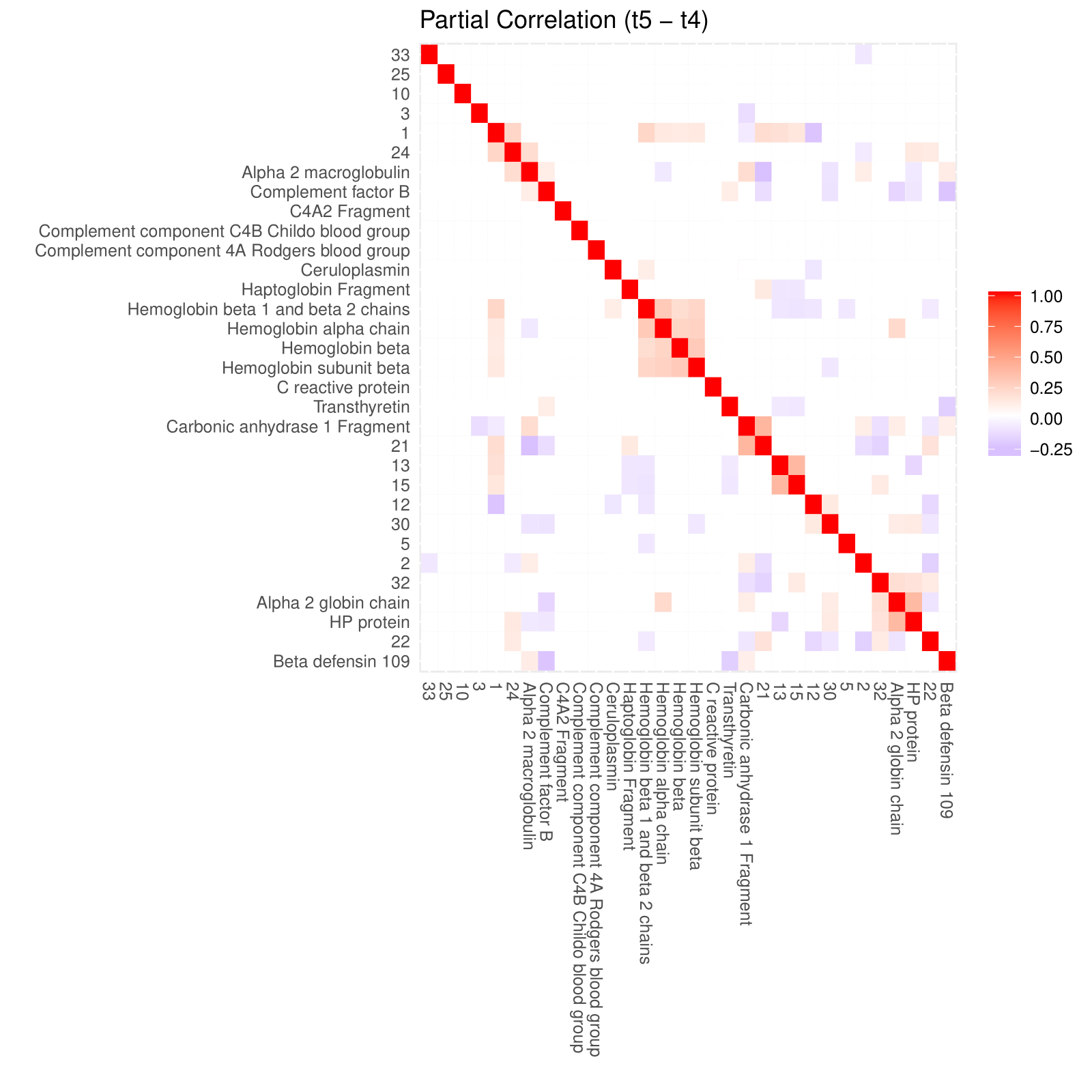

Supplement: S25 Fig — Row and column order was set by hierarchical clustering using 1−cor(x), or 1−pcor(x) as distance function for the marginal and partial correlations, respectively. Color represents positive (red) or negative (blue) correlations. (TIFF) [file pone.0222403.s025.tiff]
